# Immobilizing amine species in a Cr-MOF for enhanced selectivity in CO_2_ cycloaddition reactions†

**DOI:** 10.1039/d4cc05453a

**Published:** 2025-04-01

**Authors:** Anita Justin, Till Schertenleib, Jocelyn Roth, Jordi Espín, Wendy L. Queen

**Affiliations:** a Institute of Chemical Sciences and Engineering (ISIC), Laboratory for Functional Inorganic Materials (LFIM), École Polytechnique Fédérale de Lausanne (EPFL) Lausanne1015 Switzerland wendy.queen@epfl.ch

## Abstract

After grafting polyalkylamines into a Cr^3+^-MOF, the materials were tested for their efficacy in catalyzing cycloaddition reactions between CO_2_ and 1,2-epoxybutane (EB). While the amines significantly increased the MOF's cyclic carbonate selectivity, Cr^3+^ promoted the formation of unwanted polymeric side-products that became trapped in the MOF pores, reducing catalyst cyclability.

## Abstract 2

After grafting polyalkylamine species onto the ligand within a Cr^3+^-MOF, the material was tested for catalyzing cycloaddition reactions between CO_2_ and 1,2-epoxybutane (EB).

## Graphical abstract

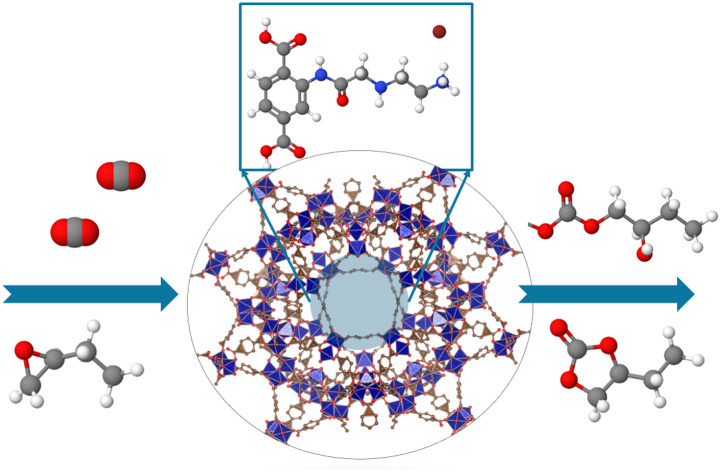

Among many classes of heterogeneous, CO_2_ conversion catalysts, metal–organic frameworks (MOFs) stand out due to their tuneable pore sizes and shapes, exceptional internal surface areas (SA), and the ease with which catalytically active sites can be precisely engineered into their pores.^1^ While MOFs have been employed in the catalytic conversion of CO_2_ into many chemicals and fuels like formic acid, methanol, ethanol, methane, *etc.*,^2^ cycloaddition reactions, used to form cyclic carbonates (CCs), are particularly prominent in the literature.^3^ This attraction stems from the rather mild reaction conditions, which are conducive to MOF stabilities, and the broad use of CCs as polar solvents, electrolytes in batteries, and precursors for pharmaceuticals and polymers, such as polycarbonates and polyurethanes.^4^ Further, benchmark methods used to produce CCs rely on toxic and corrosive phosgene,^5^ highlighting the need for more sustainable processes.

CO_2_ cycloaddition reactions typically involve a Brønsted or Lewis acid (LA) site that activate an epoxide precursor, which is then attacked by a Lewis base (LB), such as a tetraalkylammonium halide, forming a haloalkoxide intermediate. This intermediate reacts with CO_2_ to produce the desired CC.^3^ As MOFs naturally consist of LA metal units, co-catalysts providing LB functionality are often introduced for cycloaddition reactions.^6^ Recent efforts in the field have emphasized the post-synthetic introduction of LB groups like amines, quaternary ammonium salts, and ionic liquids directly in the MOF pores^6^ to enhance the catalytic efficiency of the materials. For example, Ding *et al.* crosslinked 1-vinyl-3-ethylimidazolium bromide (VEIMBr) and *ortho*-divinylbenzene (*o*-DVB) within MIL-101(Cr), achieving CC formation at 1 bar CO_2_.^7^ Further, imidazolium-based ionic liquids (ILs) were incorporated into MIL-101(Cr) and MIL-101(Cr)-CH_2_Cl. The IL was covalently grafted to the ligand in CH_2_Cl-MIL-101(Cr) and coordinated to Cr^3+^ in MIL-101(Cr)-CH_2_Cl.^8^ Importantly, covalent grafting ensured stable catalytic performance over five cycles, whereas coordination to Cr^3+^ led to IL loss, reducing catalyst cyclability. Similarly, quaternary ammonium salts were grafted to ligands in NH_2_-MIL-101(Cr) for CO_2_ cycloaddition reactions.^9,10^ These studies all reported high conversion and selectivity and catalyst stability due to the grafted co-catalysts.

We recently developed a strategy to covalently graft small alkyl amines into MOF pores for CO_2_ capture applications.^11,12^ After grafting (Scheme 1), some of the amines remain protonated leaving behind protic amine salts (NH_3_^+^Br^−^). Knowing the previous literature, it was hypothesized that such modified MOFs may perform well in CO_2_ cycloaddition reactions due to the presence of LA metals (Cr^3+^) and multiple LBs including amines (–NH_2_, –NH–) and protic amine (NH_3_^+^Br^−^) salts.

**Scheme 1:**
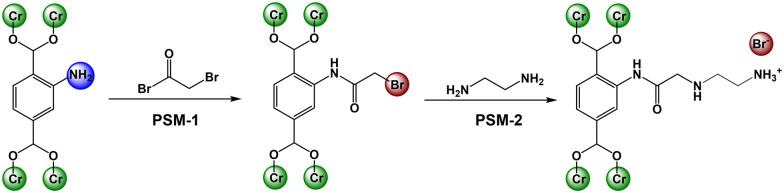
Post-synthetic modification of NH_2_-Cr-BDC with bromoacetyl bromide (BrAcBr) and then amines (ED).

Thus, in the present study, three amines, including ethylene diamine (ED), diethylene triamine (DETA) and tris(2-aminoethyl)amine (TAEA), were covalently grafted to the NH_2_-BDC ligand in a robust Cr-MOF, known as NH_2_-Cr-BDC or NH_2_-MIL-101(Cr)^13^ and tested in cycloaddition reactions.^11,12^ For this, the MOF, constructed from 2-aminoterephthalic acid (NH_2_-BDC) and Cr_3_(μ^3^-O) clusters, was made using previously reported procedures^14^ and found to be crystalline and pure (Fig. S1a, ESI†). The average particle size was 35(5) nm (Fig. S1c, ESI†). After solvent removal, the BET SA and pore volume were ∼2200 m^2^ g^−1^ and 1.49 cm^3^ g^−1^, respectively (Fig. S1b, ESI†).

After MOF characterization, the post-synthetic modification (PSM) was done in two steps (Scheme 1, see ESI,† for details).^12^ In the first step, PSM-1, the MOF was treated with bromoacetyl bromide (BrAcBr), converting the NH_2_-BDC ligand to BrAc-NH-BDC (Scheme 1). Successful grafting was supported *via* stretching vibrations observed at 1705 cm^−1^ and 3295 cm^−1^ in the FTIR data, revealing the formation of carbonyl (–C=O) groups and secondary amines (–NH–), respectively (Fig. S2a and b, ESI†). Further, XPS data of the Br 3d region revealed peaks at BEs (binding energies) of 70.64 and 71.7 eV (3d_5/2_ and 3d_3/2_, respectively) representative of C–Br bonds (Fig. S2c, ESI†) and thus, successful grafting.^12^ Two other Br species were also present, including Cr–Br and NH_3_^+^Br^−^ at 68.27 and 69.32 eV (3d_5/2_ and 3d_3/2_), respectively.^12^ Although, the two are indistinguishable *via* Br-3d XPS, N 1s XPS^15^ reveals peaks at 399.12 eV, 400.12 eV and 401.28 eV, which are assigned to NH_2_, –NH–C=O, and NH_3_^+^Br^−^, respectively, supporting the presence of the protonated salts (Fig. S2d, ESI†). Quantification of Br^−^ species *via* XPS, indicates ∼60% C–Br (organic Br) and ∼40% inorganic Br (Cr–Br/NH_3_^+^Br^−^) (Table S1, see ESI,† for details).

Next, for PSM-2, BrAc-NH-Cr-BDC was treated with three different amines, including ED, DETA, and TAEA. The materials are denoted [amineH^+^Br^−^]-Ac-NH-Cr-BDC. The XPS of the Br 3d region revealed the absence of C–Br peaks confirming successful conversion with amines. The peaks observed at 67.77 and 68.84 eV (3d_5/2_ and 3d_3/2_, respectively) indicate that the inorganic Br^−^ species remain in the framework (Fig. 1a),^12^ and the BE at 401.07 eV in the N 1s region confirms the presence of protonated amine salts, NH_3_^+^Br^−^ (Fig. 1b).

**Fig. 1:**
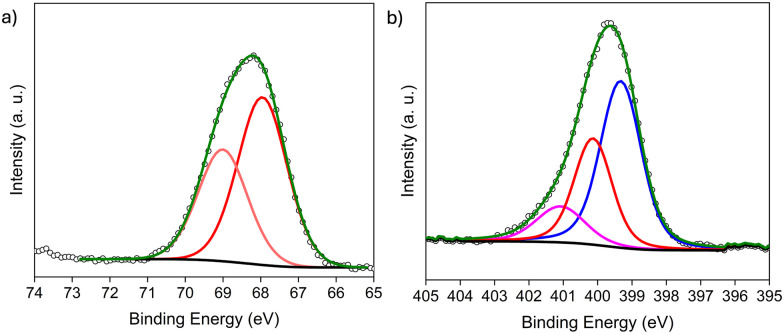
XPS data of the (a) Br 3d region and (b) N 1s region of [TAEAH^+^Br^−^]-Ac-NH-Cr-BDC after PSM-2. Colors and shapes represent Br^−^ (red), –NHC=O, amide (red), –NH_2_ (blue), NH_3_^+^Br^−^ (magenta), and the background (black), fit (olive) and data (black hollow spheres).

The SA, pore volume, Br and N content, and mass% of organics in the MOF pores are shown for each composite after PSM-1 and PSM-2 (Table S2, ESI†). The total Br content in the composites determined *via* ion chromatography (IC) expectedly decreased from ∼23 wt% (after PSM-1) to ∼5.0 wt% (after PSM-2) (Table S2, ESI†). Further, combustion analysis indicated a significantly higher N content after PSM-2, further supporting successful incorporation of amine species in the MOF pores (Table S2, ESI†). The amine-grafted frameworks were all crystalline (Fig. S3, ESI†), and the BET SA, which dropped after PSM-2, correlate directly with the size of grafted amine (Table S2 and Fig. S4, ESI†). Thermal gravimetric analysis (TGA) indicates a larger organic content in the MOF after amine grafting (Table S2 and Fig. S5, ESI†).

Given the addition of Lewis base functionality (–NH_2_, –NH–, Br^−^) after PSM-2, the composites were subjected to CO_2_ cycloaddition reactions (1 or 5 bar and 70 °C for 26 h) with 1,2-epoxybutane (EB) and their performance was compared to the parent MOF. The aim was to assess the effect of the different grafted amine salts on the conversion, selectivity, and the reusability of the catalyst. The first catalytic tests were performed at 1 bar CO_2_ and analysed *via*^1^H-NMR. In all cases, the ^1^H-NMR spectra (Fig. S6–S10, ESI†) of the supernatant revealed at least three components, including unreacted EB, the desired CC and polymeric byproducts, which could consist of a polycarbonate, polyepoxide, or a co-polymer of both (Fig. S6, ESI†). Proton signals from each species, were used to determine the conversion rate (based on EB) and selectivity of CC over the undesired products (see ESI† Eqn S1 and S2 for details). Importantly, the bare MOF, NH_2_-Cr-BDC, without Br^−^, promoted 100% EB conversion, but only with 37(1)% CC selectivity, indicating that much of the EB goes towards the formation of the polymeric byproducts (Fig. 2a and Fig. S7, ESI†). This stems from the presence of Cr^3+^ or –NH_2_ groups that can facilitate the cycloaddition reaction.^7,16,17^ MALDI-MS taken from the supernatant revealed that the polymeric species have a maximum *m*/*z* of 742 (Fig. S11, ESI†) and that the polymers consist of epoxide and CO_2_ (structure in Fig. S11, ESI†) building blocks as several peaks have increments of 72 (EB) or 116 (EB + CO_2_).

**Fig. 2:**
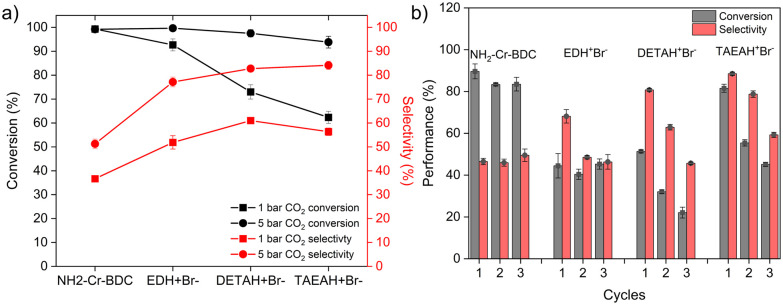
Catalysis data including: (a) conversion (black) and selectivity (red) of NH_2_-Cr-BDC and amine composites in 1 bar (solid square) and 5 bar (solid circle) CO_2_ and (b) the cycling data of the materials in 5 bar CO_2_ for 6 h.

Like NH_2_-Cr-BDC, CC and the polymeric species were analyzed in the reaction mixture of all three [amineH^+^Br^−^]-Ac-NH-Cr-BDC composites (Fig. S6–S10, ESI†). There is a trend in EB conversion, which follows [EDH^+^Br^−^]-Ac-NH-Cr-BDC > [DETAH^+^Br^−^]-Ac-NH-Cr-BDC > [TAEAH^+^Br^−^]-Ac-NH-Cr-BDC (Fig. 2a and Table S3, ESI†). This could imply that the smaller ED (0.26 nm radius) and/or overall higher SA (1260 m^2^ g^−1^) of [EDH^+^Br^−^]-Ac-NH-Cr-BDC could allow more efficient diffusion of EB into the MOF pores. Likewise, the bulkier TAEA (0.46 nm radius) and lower overall SA (780 m^2^ g^−1^) of [TAEAH^+^Br^−^]-Ac-NH-Cr-BDC may hinder EB diffusion. Surprisingly, the substrate selectivity fell within a narrow range, 52(3)–61(2)%, for all amine-grafted materials indicating selectivity may not be significantly impacted by the amine structure (Fig. 2a and Table S3, ESI†). The selectivity of all amine-grafted materials was however higher than NH_2_-Cr-BDC, 37(1)%. This could stem from the presence of amineH^+^Br^−^ salts or alkyl amines that better facilitate CO_2_ activation (CO_2_^δ−^ adducts or carbamates, respectively)^11^ and subsequent cycloaddition of the epoxide. Interestingly, the polymer species found in the supernatant of [EDH^+^Br^−^]-Ac-NH-Cr-BDC were also analysed *via* MALDI-MS and found to have a *m*/*z* up to 1018 (Fig. S12, ESI†). This *m*/*z* value is larger than the one observed for NH_2_-Cr-BDC, potentially indicating that the grafted amine species in the MOF pores may facilitate the formation of polymers having higher molecular weights.

Given the formation of polymeric species, we also assessed the catalytic activity of Cr-BDC, also known as MIL-101 (Cr), in the same reaction. A previous report indicated that this MOF could promote similar cycloaddition reactions, despite the absence of Lewis basic NH_2_ sites; however, no polymer side products were reported.^7,17^ Thus, our goal was to see if Cr^3+^ plays a role in the polymer formation. Interestingly, Cr-BDC facilitated 100% conversion of EB with 60(6)% selectivity towards CC with the formation of polymeric side products (Fig. S13, ESI†). Next, several control reactions were performed to investigate the polymer products. First, NH_2_-Cr-BDC, [EDH^+^Br^−^]-Ac-NH-Cr-BDC, and Cr-BDC were tested under the same reaction conditions but excluding CO_2_. Surprisingly, polymer signals were observed in ^1^H-NMR (3.25–3.75 ppm) of poly-EB in all three materials (Fig. S14, S15 and S16a, ESI†). The MALDI-MS revealed poly-EB with *m*/*z* values equivalent to ∼8 units, ∼9 units, and ∼12 units for Cr-BDC, NH_2_-Cr-BDC, and [EDH^+^Br^−^]-Ac-NH-Cr-BDC, respectively (Fig. S16b and S17, ESI†). This implied that Cr^3+^ alone facilitated polymer formation and showed that basic groups, like alkylamines, more readily formed higher molecular weight polymers. To eliminate the possibility of self-polymerization of EB to poly-EB, a control reaction was performed containing only EB with no MOF or CO_2_, in ACN at 70 °C for 26 h; however, no polymerization was observed (Fig. S18, ESI†). While a recent report showcased the ability of a Cr^3+^ complex to polymerize cyclohexane oxide to poly-cyclohexane oxide,^18,19^ to the best of our knowledge, similar polymerization reactions have not been observed in Cr-MOFs.

Given the intrinsic ability of Cr^3+^ to polymerize EB and CO_2_ into a co-polymer side product, several reaction conditions were altered to enhance selectivity and minimize unwanted polymeric byproducts during cycloadditions. For example, reactions were carried out at lower EB concentrations, shorter reaction times, and/or lower reaction temperatures (Fig. S19, ESI†). However, these efforts led to minimal to no increase in selectivity, suggesting that polymerization between the epoxide and CO_2_ occurred concurrently with CC formation. In a last attempt, the CO_2_ pressure was increased from 1 bar to 5 bar to improve the solubility of CO_2_ and hopefully push the conversion towards the CC while suppressing the polymerization of EB.^20^ As expected, the conversion was boosted close to ∼90–100% for all composites and selectivity increased to 77(2) to 84(2)% for all three amine-grafted catalysts (Fig. 2a and Fig. S20–S23, ESI†). For the parent MOF, NH_2_-Cr-BDC, the selectivity was improved to 51(3)%, albeit the increase is much smaller than that of the amine-grafted materials (Fig. 2a and Table S4, ESI†). In no case was 100% selectivity achieved as the formation of polymers was found to be unavoidable, likely due to the presence of Cr^3+^ (Fig. 2a and Fig. S20–S27, ESI†).

Next, catalyst recyclability was assessed at 5 bar CO_2_ for 6 h at 70 °C over 3 cycles (Fig. 2b). After each cycle, the catalysts were washed, and after the third cycle, the spent catalysts were also vacuum dried before collecting FTIR, TGA and PXRD data. For NH_2_-Cr-BDC, there was only a slight drop in EB conversion from 89(4) % to 83(3) % over 3 cycles, and the selectivity was maintained at ∼45(2) % to 49(3) % (Fig. 2b and Fig. S28–S30 and Table S5, ESI†). FTIR data were also collected on the spent NH_2_-Cr-BDC catalyst before and after washing with acetonitrile. The unwashed catalyst had an intense band at ∼1780–1800 cm^−1^ corresponding to carbonyl (–C=O) stretching of CC, a less intense band at ∼1745 cm^−1^ due to –C=O stretching of entrapped polycarbonate co-polymers, and an IR band at ∼1060 cm^−1^ corresponding to –C–O–C– stretching of ether linkages of entrapped polymers (Fig. 3a).^21^ However, for the washed NH_2_-Cr-BDC catalyst, only the peak at ∼1745 cm^−1^ remained, indicating that the washing step removed the CC product and likely the low molecular weight polymers (Fig. 3a). Further, the shift in the –NH_2_ stretching vibration of NH_2_-Cr-BDC to lower wavenumbers after the catalytic cycles likely indicates H-bonding interactions between the entrapped polymers and amino ligands, inhibiting their full removal (Fig. 3b). While the TGA revealed an accumulation of organics, ∼22 wt% (Fig. 3c), the conversion and selectivity of the parent NH_2_-Cr-BDC were not greatly impacted. This is surprising considering the huge drop in the SA and (1140 m^2^ g^−1^ and 0.91 cm^3^ g^−1^, respectively) after the first run (Fig. S31a, S32a and Table S6, ESI†). However, it indicates that the substrates could still access the active sites (Cr^3+^ and amine species).

**Fig. 3:**
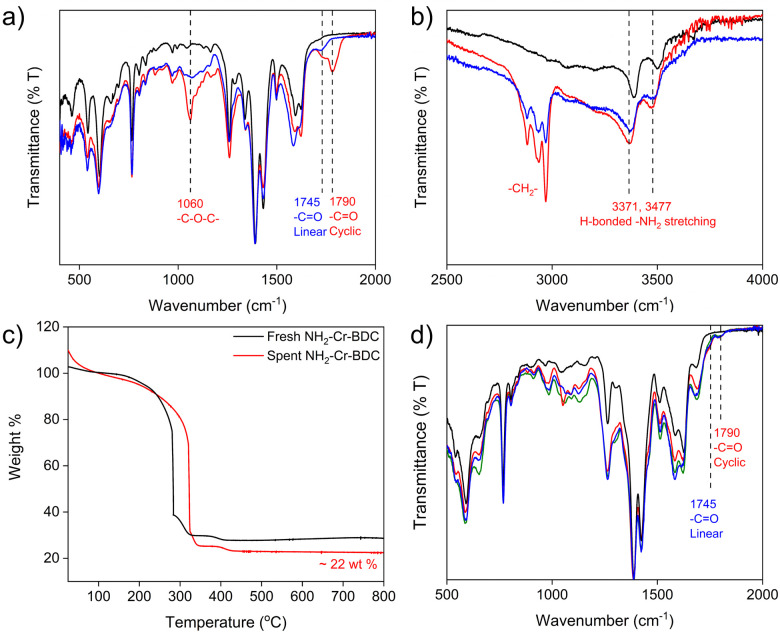
FTIR data of fresh (black) and spent NH_2_-Cr-BDC before (red) and after washing (blue) in the range of (a) 500–2000 cm^−1^ and (b) 2500–4000 cm^−1^; (c) TGA data of fresh (black) and spent NH_2_-Cr-BDC after washing (red) and (d) FTIR data of [amineH^+^Br^−^]-Ac-NH-Cr-BDC composities including: fresh TAEA (black), spent ED (red), spent DETA (blue) and spent TAEA (green) samples after 3 cycles of catalysis.

Relative to the parent NH_2_-Cr-BDC, the conversion and selectivity of the catalysts containing grafted amine species declined more significantly with cycling, though their overall selectivity remained similar or superior to NH_2_-Cr-BDC (Fig. 2b and Fig. S33–S41, Table S5, ESI†). Various forms of characterization were employed to understand the performance decline. While PXRD confirmed that all catalysts maintained structural integrity with cycling (Fig. S42–S45, ESI†), FTIR spectra revealed distinctive carbonyl stretching vibrations at 1745 cm^−1^ for a polycarbonate co-polymer and at ∼1780–1800 cm^−1^ for CC, even after extensive catalyst washing (Fig. 3d).^21^ This indicated retention of organics in the MOF pores, stabilized through H-bonding interactions with grafted amines that hindered the removal of the low molecular weight species (Fig. 3b). The presence of such species, which likely limited access to active sites, was further supported by significant decreases in both the SAs and pore volumes of the composites after catalysis (Fig. S31, S32 and Table S6, ESI†). Also, TGA data taken from the spent and fresh catalysts confirmed significant entrapment of byproducts with increases in organic content (amines + catalytic byproduct) of ∼48, 44, and 49 wt% for [EDH + Br^−^]-Ac-, [DETAH + Br^−^]-Ac-, and [TAEAH + Br^−^]-Ac-NH-Cr-BDC, respectively, after catalysis. Importantly, these values are approximately double that observed in the parent NH_2_-Cr-BDC (Fig. 3c and Fig. S46–S48, ESI†). While all data suggests that the retention of byproducts is the likely culprit for the performance decline of the amine-grafted composites, we also noted Br^−^ leaching during catalysis *via* IC (Table S6, ESI†). However, since protonated and non-protonated amine species both play a role in the reaction, it is still uncertain if Br^−^ loss may also contribute to the composites’ declining catalytic performance.

While this work showed that grafting amines and/or protic amine salts (–NH_3_^+^Br^−^) into MOF pores can enhance selectivity for a desired cyclic carbonate during catalytic CO_2_ cycloaddition reactions, it was also found that Cr^3+^ drives the formation of unwanted polymeric byproducts. Although Cr-MOFs were employed in similar reactions before, the formation of these byproducts was missed, likely leading to exaggerated conversion rates. Moreover, the grafted amine species were found to promote the formation of higher molecular weight polymers that become more readily trapped inside the Cr-MOF pores. In cycling experiments, this leads to a faster catalyst degradation when compared to the unmodified parent MOF, NH_2_-Cr-BDC. Despite their catalytic activity, Cr^3+^-based materials may present inherent limitations for cycloaddition reactions due to their promotion of unwanted polymerization reactions. Thus, future investigation of catalysts containing alternative metal centres are warranted.

The work was partially supported by the Swiss National Science Foundation under grant number 200021_188536.

## Data availability

The data supporting this article have been included as part of the ESI.† Raw data files for all the plots in the manuscript and the ESI,† are available on Zenodo at https://doi.org/10.5281/zenodo.15035458.

## Conflicts of interest

There is no conflict to declare.

## Supplementary Material

CC-061-D4CC05453A-s001
