# Supplementary material for: Immobilizing amine species in a Cr-MOF for enhanced selectivity in CO2 cycloaddition reactions
Source: Chem Commun (Camb). 2025 Apr 1;61(35):6530–3. doi: 10.1039/d4cc05453a (PMC11973883; doi:10.1039/d4cc05453a)
Supplement: CC-061-D4CC05453A-s001 [file CC-061-D4CC05453A-s001.pdf]

Supplementary Information (SI) for Chemical Communications.
This journal is © The Royal Society of Chemistry 2025

Supplementary Information

Immobilizing Amine Salts in a Cr-MOF for Enhanced Selectivity in CO_2 Cycloaddition Reactions

*Anita Justin,[a] Till Schertenleib,[a] Jocelyn Roth Richard,[a] Jordi Espin,[a] Wendy L. Queen[a] **

a Institute of Chemical Sciences and Engineering (ISIC), Laboratory for Functional Inorganic Materials (LFIM), École Polytechnique Fédérale de Lausanne (EPFL), Laboratory for Functional Inorganic Materials (LFIM), Sion, Switzerland

Keywords: Metal-Organic Frameworks, Post-synthetic modification, Protic amine salts, Covalent grafting, Cyclic carbonate, Polymers, CO_2 Cycloaddition.

Table of Content

Section S1: Characterization techniques S3

X-ray Diffraction (XRD) S3

Electron Microscopy (EM) S3

Ion Chromatography (IC) S3

Elemental Analysis (EA) S3

MALDI-MS S3

Microwave Digestion S3

X-ray Photoelectron Spectroscopy (XPS) S3

Nuclear Magnetic Resonance (NMR) Spectroscopy S4

Thermogravimetric Analysis (TGA) S4

Nitrogen Adsorption S4

Section S2: Materials and Methods S6

Section S3: Characterizations S8

Section S1: Characterization techniques

X-Ray Diffraction:

X-ray diffraction (XRD) patterns were performed on a Bruker D8 diffractometer with Cu Kα radiation (λ= 1.5418 Å) at 40 kV and 40 mA. Powder samples were placed on the sample holder for X-ray diffraction patterns.

Electron microscopy:

Bright-field transmission electron microscopy (TEM) images were taken using the Thermo Scientific Tecnai G2 Spirit Twin instrument at an accelerating voltage of 120 kV. High-angle annular dark-field scanning transmission electron microscopy (HAADF-STEM) imaging was performed on a double Cs-corrected Thermo Scientific Titan Themis instrument operated in scanning mode at an accelerating voltage of 200 kV. This microscope is equipped with a high brightness X-FEG gun and four silicon drift Super-X EDX detectors.

Ion Chromatography (IC):

Amount of bromide anion was quantified with Thermo Scientific Dionex Integrion HPIC System, equipped with the AS-AP autosampler, EG, CR-TC, conductivity detector, suppressor and the Dionex IonPac AS15 column and pre-column. Samples were analyzed according to the routine procedure for the detection of common anions: 38 mM KOH, 0.35 mL/min flow rate, 30 º C. The instrument was calibrated using 7 Anion standards of Thermo Scientific.

Elemental Analysis (EA):

EA tests were carried out using a Unicube instrument from Elementar.

MALDI-MS:

Matrix-assisted laser desorption/ionization mass spectrometry (MALDI-MS) analysis was performed using a Bruker Microflex. The mode was set to linear with a positive polarity.

Microwave digestion:

The samples, 2-5 mg, were soaked in 8 M NaOH solution and digested using microwave oven (synthWave, MWS Mikrowelle GmbH) pressurized with N_2 (initial pressure of 20 bars). Digestion program was the following: 5 min to heat up to 120°C, then 5 min to heat up to 150°C and keep at 150°C for 15 min.

X-ray Photoelectron Spectroscopy (XPS):

X-ray photoelectron spectroscopy (XPS) measurements were carried out on a Kratos AXIS Supra instrument with a Al Kalpha X-ray source. Data was fitted using CasaXPS. All spectra were referenced to the C-C peak at 284.8 eV. The Br 3d doublets were constrained with a peak splitting of 1.05 eV and a relative peak area of 3:2 for (5/2):(3/2). The full-width-half-maximum of the peaks were all constrained to be equal. The C 1s, O 1s, N 1s, Br 3d, and Cr 2p peaks were integrated and compared for the relative atomic

percentage quantification. Percentage contribution of C-Br and Br^- is calculated from the area under corresponding BE divided by total area (Sum of all Br BE) of Br 3d.

Nuclear Magnetic Resonance (NMR):

Solution NMR spectra were recorded in deuterated solvents on a BRUKER AVIII HD 400 spectrometer; data are given in ppm relative to 1% TMS solution in $CDCl_3$. NMR spectra were recorded at room temperature, if not mentioned otherwise. The Bruker Topspin software package (version 3.2) was used for measuring and Mestrenova NMR software (version 11.0.1) was used for processing of the spectra. Conversion rate were calculated by the following equations,

$$\text{Equation S1: Conversion} = \frac{\text{Hc}' + \text{Hd}''/2}{\text{Hc} + \text{Hc}' + \text{Hd}''/2} \text{ x } 100\ \%$$

Equation S1: formula for total conversion of EB to cyclic carbonate and polycarbonate copolymer

$$\text{Equation S2: Selectivity} = \frac{\text{Hd}'}{\text{Hd}' + \text{Hd}''} \text{ x } 100\ \%$$

Equation S2: Formula for calculating selectivity for the cyclic carbonate over other polymeric products, otherwise known as cyclic carbonate selectivity.

Note: The selectivity we obtained from NMR (Eq. 2) outlines the selectivity for the cyclic carbonate, which is calculated based on the formula,

$$CC\ Selectivity\ = \frac{\text{mol of desired product (CC)}}{(mol\ of\ starting\ reagent\ (EB) - mol\ of\ reagent\ at\ the\ end\ of\ reaction\ (EB))}$$

Since only CC and polymeric byproducts were detected, it is assumed that the remainder of the EB goes towards the formation of poly-epoxides and/or co-polymers.

Thermogravimetric analysis (TGA):

For TGA runs from 25 to 800 ºC, we used a Q500 instrument from TA under air flow. Approximately 5-10 mg of sample was employed in each test. After each run, the TGA curve is normalized to 120 ºC to subtract the contribution from solvents. The residual weight, that is the weight % at 800 ºC was used to calculate the organic content in the MOF composites.

$$Equation\ S3\text{: } organic\ wt\ \%\ = 1 - \frac{\text{Residual wt\% of amine MOF composites}}{\text{Residual wt\% of bare MOF}}$$

Equation S3: Formula for calculating organic wt % in the amine MOF composites.

Nitrogen Adsorption:

For nitrogen adsorption measurements 80 - 100 mg of Bare MOF and modified MOF was transferred to a pre-weighed glass sample tube under an atmosphere of nitrogen and capped. Samples were then transferred to the activation station Belsorp vac II and placed in vacuum at r.t for 24 h for complete evacuation. Samples were considered activated when the outgas rate at 298 K was less than 2 µbar/min. Evacuated tubes containing degassed samples were then transferred to a balance and weighed to determine the mass of

sample, typically 60 – 90 mg. The tube was transferred to the analysis port of the instrument Belsorp max. Nitrogen gas adsorption isotherms at 77 was measured in liquid nitrogen.

Materials and Methods

All chemicals were obtained from commercial sources and used as received without further purification. Chromium nitrate nonahydrate, $Cr(NO_3)_3.9H_2O$ (Acros, 98 %), 2-aminoterephthalic acid, NH_2-BDC (ABCR, 98 %), bromoacetyl bromide, BrAcBr (ABCR, 98 %), tetrahydrofuran, THF (Roth, 99.5 %), N,N-dimethylformamide, DMF (Roth, 99.5 %), Acetonitrile, ACN (Roth, 99.5 %) 1,2-Epoxybutane (Sigma Aldrich, 99 %), ethylenediamine, ED (TCI, 98 %) diethylenetriamine, DETA (TCI, 98 %), tris(2-aminoethyl)amine, TAEA (ABCR, 98 %), CDCl3-*d* (Sigma Aldrich, 99.5 % D), D_2O (Sigma Aldrich, 99.5 % D) and NaOD, 40 wt % (ABCR, 99.5 % D).

Synthesis of NH_2-Cr-BDC: A 160 mL Teflon autoclave was charged with 6.4 g of $Cr(NO_3)_3.9H_2O$, 2.898 g of 2-amino-terephthalic acid (NH_2-BDC^{2-}), 1.28 g of sodium hydroxide, and 120 mL DI water. The mixture was first stirred for 15 min and sonicated for 15 min prior to transferring to a 160 mL autoclave. The oven was heated to 150 °C for 1 h at 150 °C for 12 h and cooled to 25 °C in 4 h. Once the reaction was finished, the bright green product was separated into four 50 mL centrifuge tubes and washed with 40 mL water once, 40 mL DMF thrice, and 40 mL EtOH thrice and then refluxed with 200 mL EtOH at 90 °C for 12 h two times. The resulting product was vacuum dried at RT overnight and further vacuum dried at 80 °C for 6 h. The sample was activated at 150 °C for 12 h under dynamic vacuum to access the surface area.

Synthesis of BrAc-NH-Cr-BC (PSM-1): For the first step of PSM, 110 mg of as-synthesized NH_2-MIL101(Cr) was dispersed in 5 mL THF. The temperature was adjusted to (1-4) °C with an ice bath. Then, 3 equiv. of BrAcBr with respect to the NH_2-BDC ligand was added to the cold solution and allowed to stir for 1h in the ice bath and then at RT for an additional 30 min. After the reaction, the product was washed with 20 mL fresh THF vacuum dried for spectroscopic characterization.

Synthesis of [amineH^+Br^-]-Ac-NH-Cr-BDC (PSM-2): For the second step of PSM with amines, the prepared samples after PSM-1 (without drying) was dispersed in 10 mL THF at (1-4) °C. Then, three equivalents of three different amines including ED (0.082 mL), DETA (0.125 mL) and TAEA (0.18 mL) with respect to the NH_2-BDC^{2-} were added to the cold solution and allowed to react for 1 h in the ice bath and then 30 additional min. at RT under stirring. After the reaction, the product was washed with 20 mL MeOH and vacuum dried. The sample was activated at 120 °C under dynamic vacuum to assess the porosity.

Catalysis at 1 bar CO_2 protocol: A 8 mL septum vial is charged with 20 mg catalyst, 1 mmol EB (87 µL), 0.4 mL ACN and a balloon filled with CO_2 at 1 bar pressure, under stirring at 70 °C for 26 h. Catalytic reaction was quenched in an ice-bath. The product was centrifuged, and the supernatant was collected for ^{1}H-NMR and MALDI-MS.

Control reactions protocol: A 8 mL septum vial is charged with 20 mg catalyst, 1 mmol EB (87 µL) and 0.4 mL ACN was stirred at 70 °C for 26 h. The product was centrifuged, and the supernatant was collected for ^{1}H-NMR and MALDI-MS.

Catalysis at 5 bar CO_2 protocol: A catalytic reactor of volume 20 mL is charged with 40 mg catalyst, 1 mmol EB (174 µL), 0.8 mL ACN is stirred under CO_2 pressure of 5 bar connected via CO_2 cylinder, allowed to react at 70 °C for 26 h. Prior to the reaction, the reactor is purged very slowly with CO_2 three times to

remove the air. Catalytic reaction was quenched in an ice-bath. The product was centrifuged, and the supernatant was collected for ^{1}H-NMR and MALDI-MS.

Catalyst washing protocol in catalyst reusability: A catalytic reactor of volume 20 mL is charged with 40 mg catalyst, 1 mmol EB (174 μL), 0.8 mL ACN is stirred under CO_2 pressure of 5 bar connected via CO_2 cylinder, allowed to react at 70 ºC for 6 h. After each cycle, the catalyst is washed with 20 mL fresh ACN in a vortex shaker for 30 min each to remove the regents and products. The washed catalyst is dispersed in 0.8 mL ACN for the second cycle. At the end of three cycles, the catalyst is washed and vacuum dried at RT to perform characterizations.

Section S2: Characterizations

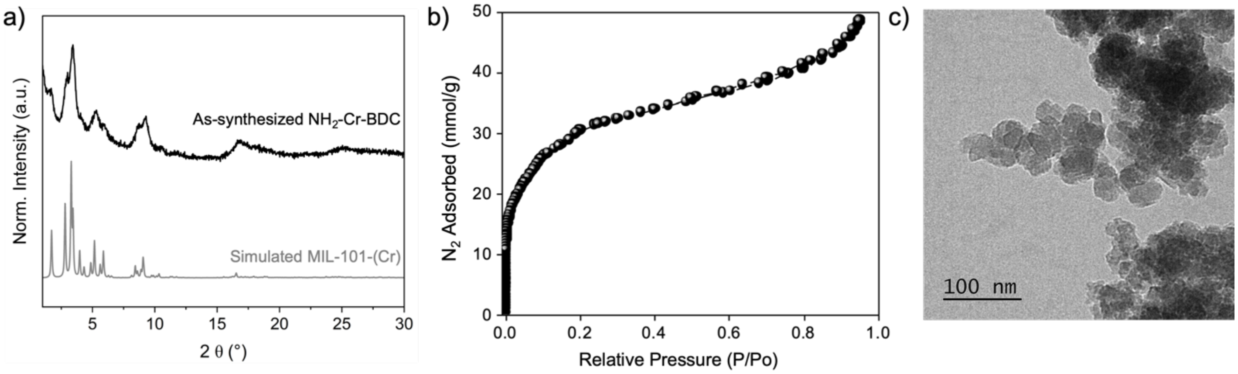

Figure S1: a) PXRD pattern of simulated pattern (grey), as-synthesized NH_2-Cr-BDC (black), b) N_2 adsorption at 77 K, c) TEM image of as-synthesized NH_2-Cr-BDC. The scale bar is 100 nm, and the average particle size is 35 ± 5.1 nm.

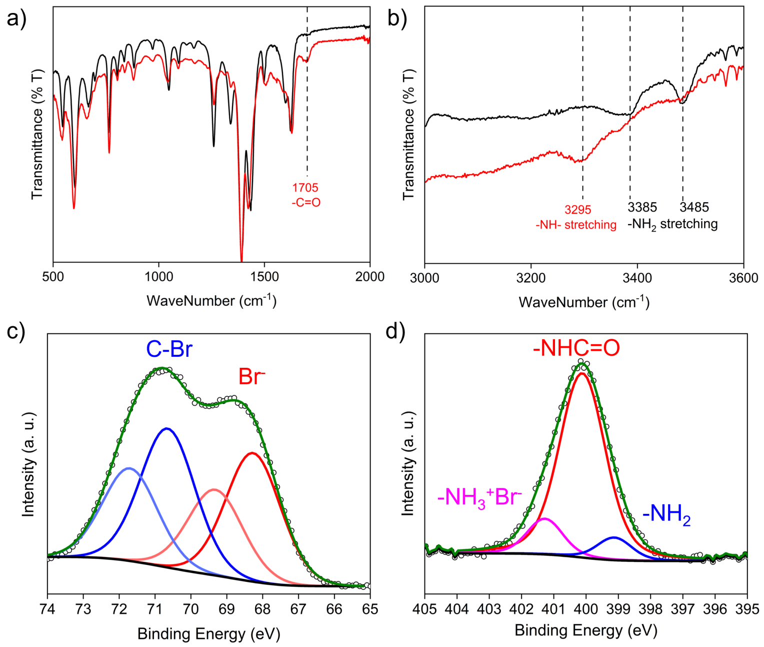

Figure S2: (a, b) FTIR spectrum of bare MOF, NH_2-Cr-BDC (black) and 3 equiv.BrAcBr treated NH_2-Cr-BDC (red). XPS of c) Br 3d region of PSM-1, d) N 1s region of PSM-1. Note: Inorganic Br, Br^- (red), C-Br (blue), -NHC=O, amide (red), $-NH_2$ (blue) and $NH_3^+Br^-$ (magenta), background (black), fit (olive) and data (black hollow spheres).

Table S1: XPS fit of Br 3d of BrAc-NH-Cr-BDC and [TAEAH$^+$Br$^-$]-Ac-NH-Cr-BDC

Composite	**Br 3d**				
BrAc-NH-Cr-BDC	Name	Cr-Br, $NH_3^+Br^-$ ($3d_{5/2}$)	Cr-Br, $NH_3^+Br^-$ ($3d_{3/2}$)	C-Br ($3d_{5/2}$)	C-Br ($3d_{3/2}$)

	Position	68.27	69.32	70.64	71.7
	FWHM	1.8	1.8	1.8	1.8
	Area	5805.61	3870.41	6267.56	4178.37
[TAEAH$^+$Br$^-$]-Ac-NH-Cr-BDC	Position	67.96	69.01		
	FWHM	1.54	1.54		
	Area	3072.24	2048.16		
Composites	**N 1s**				
	Name	N 1s NH$_2$, NH	N 1s NH-C=O	N 1s NH$_3^+$Br$^-$	
BrAc-NH-Cr-BDC	Position	399.123	400.117	401.275	
	FWHM	1.26225	1.67774	1.29779	
	Area	329.4	3565.6	534.7	
[TAEAH$^+$Br$^-$]-Ac-NH-Cr-BDC	Position	399.329	400.134	401.078	
	FWHM	1.40458	1.29287	1.66533	
	Area	4815.9	2826.1	1233	

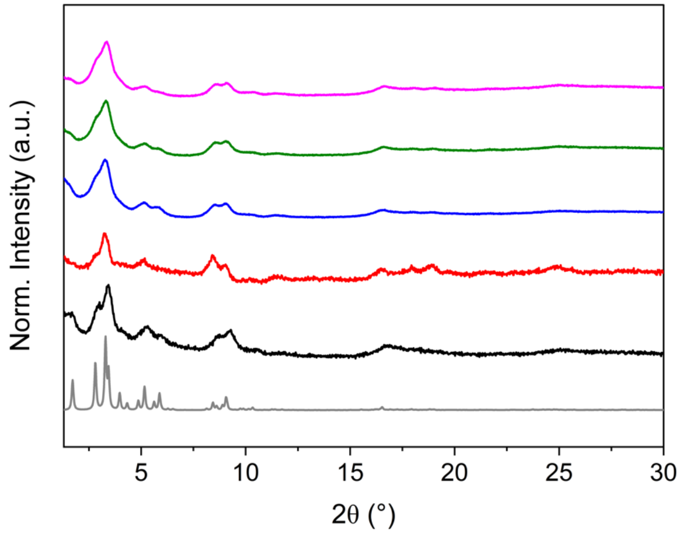

Figure S3: PXRD of simulated Cr-BDC (grey), NH$_2$-Cr-BDC (black), BrAc-NH-Cr-BDC (red), [EDH$^+$Br$^-$]-Ac-NH-Cr-BDC (blue), [DETAH$^+$Br$^-$]-Ac-NH-Cr-BDC (olive) and [TAEAH$^+$Br$^-$]-Ac-NH-Cr-BDC (magenta).

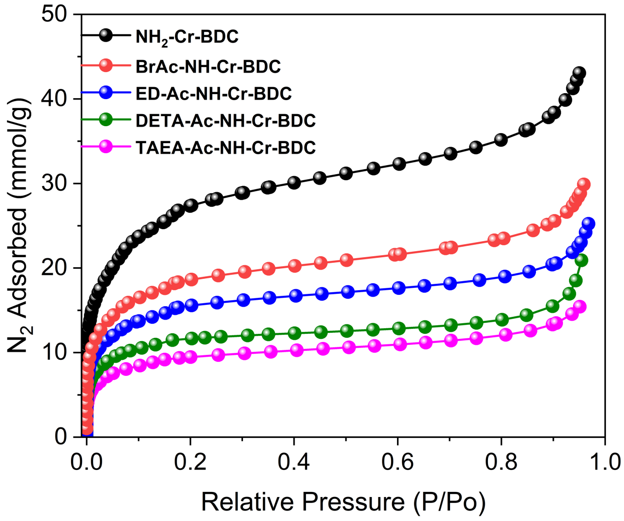

Figure S4: N_2 at 77 K of NH$_2$-Cr-BDC (black), BrAc-NH-Cr-BDC (red), [EDH$^+$Br$^-$]-Ac-NH-Cr-BDC (blue), [DETAH$^+$Br$^-$]-Ac-NH-Cr-BDC (olive) and [TAEAH$^+$Br$^-$]-Ac-NH-Cr-BDC (magenta).

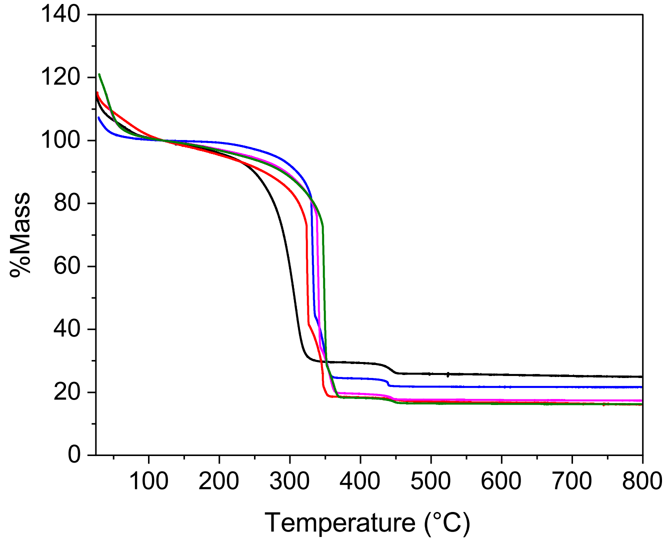

Figure S5: TGA of NH$_2$-Cr-BDC (black), BrAc-NH-Cr-BDC (red), [EDH$^+$Br$^-$]-Ac-NH-Cr-BDC (blue), [DETAH$^+$Br$^-$]-Ac-NH-Cr-BDC (olive) and [TAEAH$^+$Br$^-$]-Ac-NH-Cr-BDC (magenta).

Table S2: Summary of material characterization.

Catalyst	S_{BET} (m^2/g)	V_{pore} (cm^3/g)	Br (wt % from IC)	N (wt % from EA)	Organic loading wt %
NH$_2$-Cr-BDC	~2200	1.49	-	6.63	-
46 % BrAc-NH-Cr-BDC	1583	1.13	22.45	3.68	35.36

[$EDH^{+}Br^{-}$]-Ac-NH-Cr-BDC	1260	0.87	4.9	10.79	22.28
[$DETAH^{+}Br^{-}$]-Ac-NH-Cr-BDC	950	0.72	5.2	11.49	32.27
[$TAEAH^{+}Br^{-}$]-Ac-NH-Cr-BDC	780	0.57	5.1	12.43	34.88

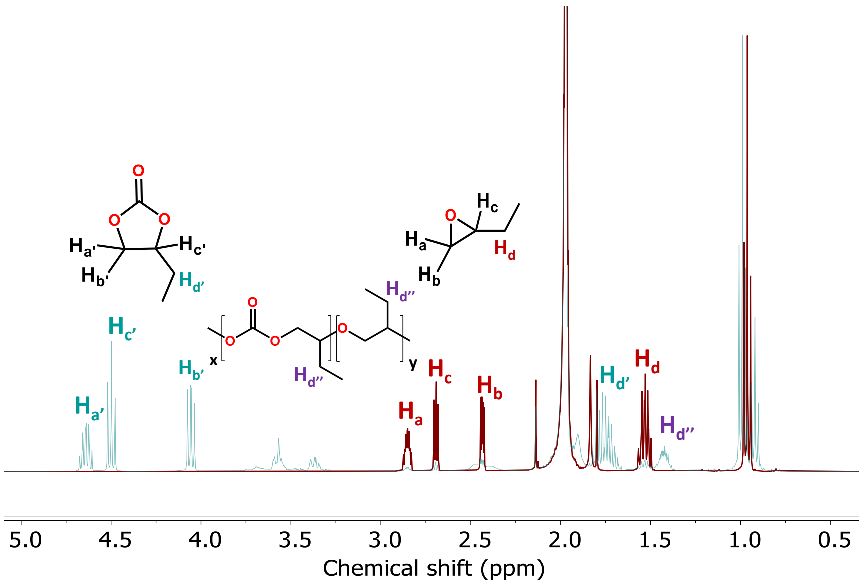

Figure S6: ^{1}H-NMR products of EB (H_a, H_b, H_c, H_d), CC ($H_{a'}$, $H_{b'}$, $H_{c'}$, $H_{d'}$) and polymer species (Hd'' and between 3.25-3.75 ppm). Proton signals between 3.8-3.2, 2.2-2.5 arise from the polymerization and side products. Proton signal between 2.2-1.8 comes from the acetonitrile solvent. Conversion of EB is calculated based on the equation S1 and selectivity is calculated based on the equation S2.

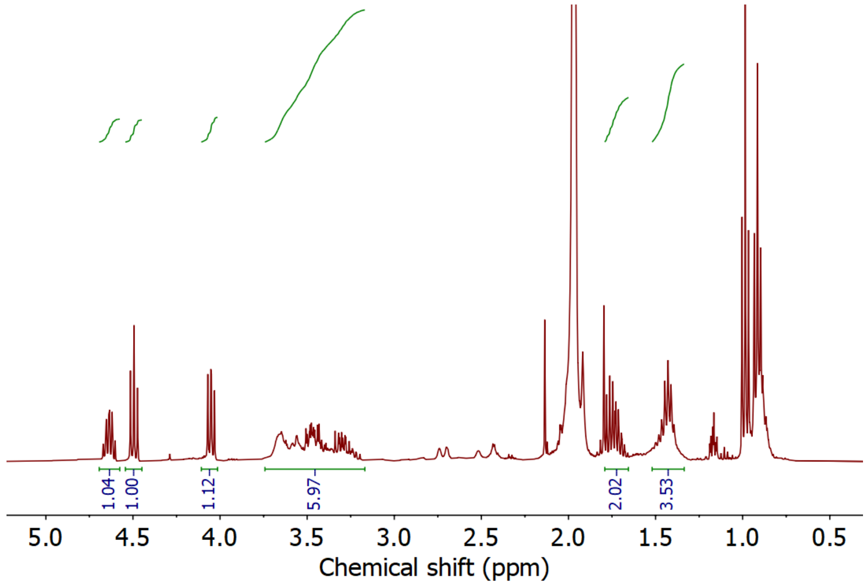

Figure S7: ^{1}H-NMR spectrum of supernatant of NH_2-Cr-BDC after catalysis in 1 bar CO_2 for 26 h.

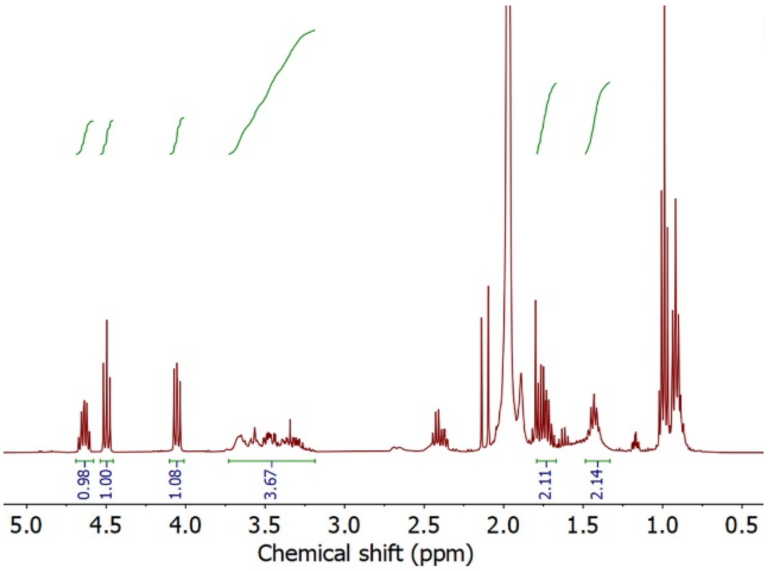

Figure S8: ^{1}H-NMR spectrum of supernatant of [EDH$^+$Br$^-$]-Ac-NH-Cr-BDC after catalysis in 1 bar CO_2 for 26 h.

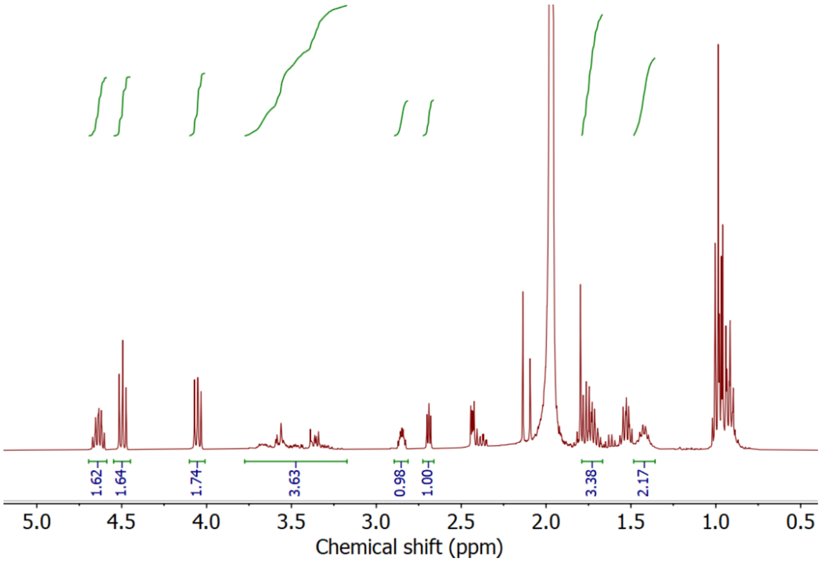

Figure S9: ^{1}H-NMR spectrum of the supernatant of [DETAH$^+$Br$^-$]-Ac-NH-Cr-BDC after catalysis in 1 bar CO_2 for 26 h.

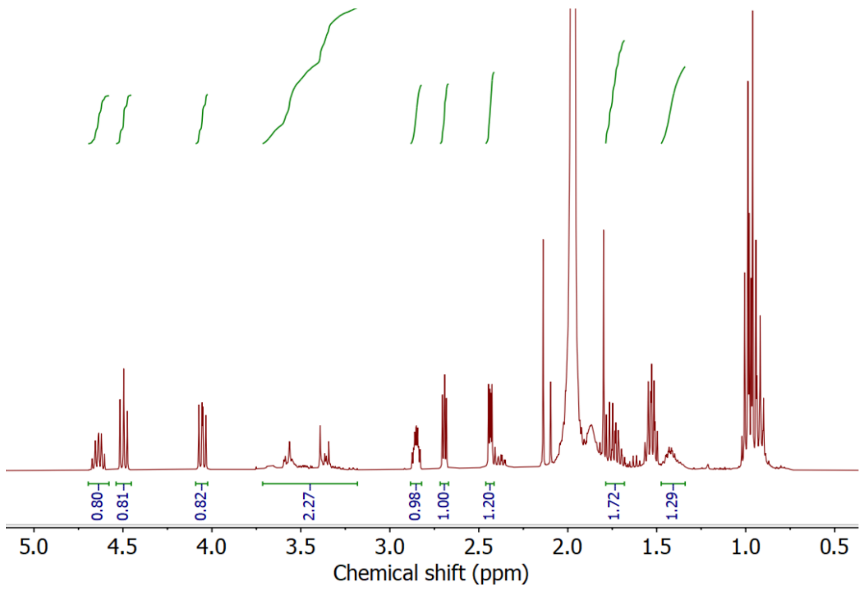

Figure S10: ^{1}H-NMR of the supernatant of of [TAEAH$^+$Br$^-$]-Ac-NH-Cr-BDC after catalysis in 1 bar CO_2 for 26 h.

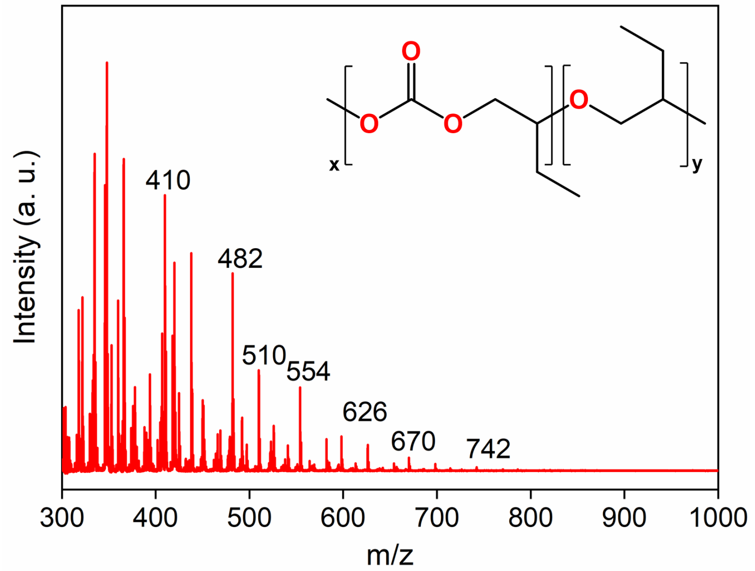

Figure S11: MALDI MS on the supernatant of NH_2-Cr-BDC after catalysis in 1 bar CO_2.

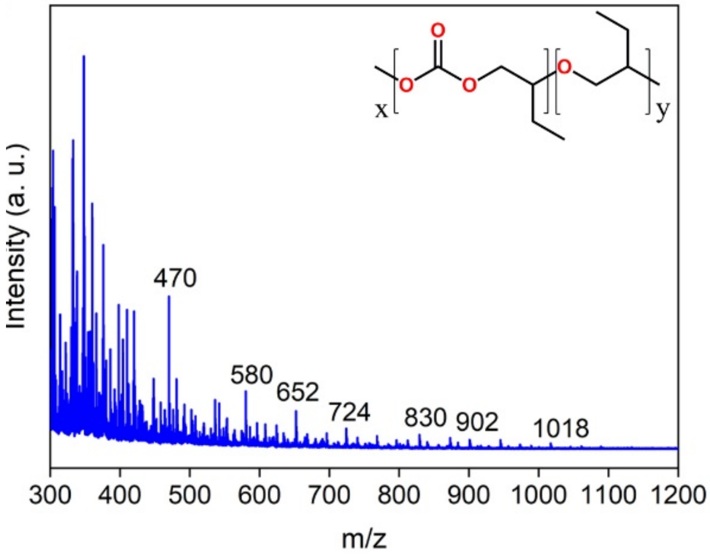

Figure S12: MALDI MS on the supernatant of [EDH$^+$Br$^-$]-Ac-NH-Cr-BDC after catalysis in 1 bar CO_2.

Table S3: Catalytic performance of materials at 1 bar CO_2, 70 °C for 26 h.

Catalysts	Conversion (%)	Selectivity (%)
NH_2-Cr-BDC	99.3 (±1.5)	36.7 (±1.2)
[EDH$^+$Br$^-$]-Ac-NH-Cr-BDC	92.7 (±2.5)	51.9 (±2.8)
[DETAH$^+$Br$^-$]-Ac-NH-Cr-BDC	73.0 (±3.0)	61.0 (±1.0)
[TAEAH$^+$Br$^-$]-Ac-NH-Cr-BDC	62.3 (±2.5)	56.3 (±1.5)

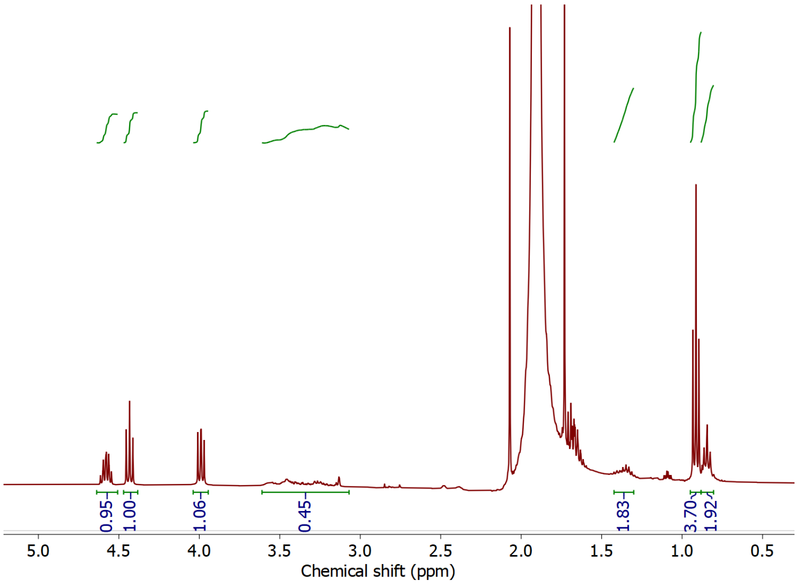

Figure S13: ^{1}H-NMR of Cr-BDC MOF after catalysis in 1 bar CO_2 for 26 h.

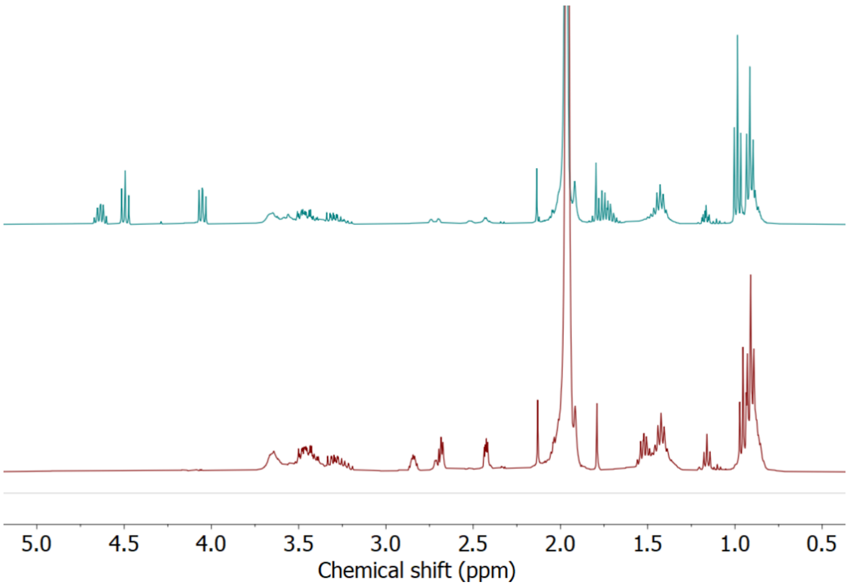

Figure S14: ^{1}H-NMR spectrum of supernatant of NH_2-Cr-BDC with EB in the absence of CO_2 for 26 h (bottom, red), reaction with EB in presence of 1 bar CO_2 for 26 h (top, teal).

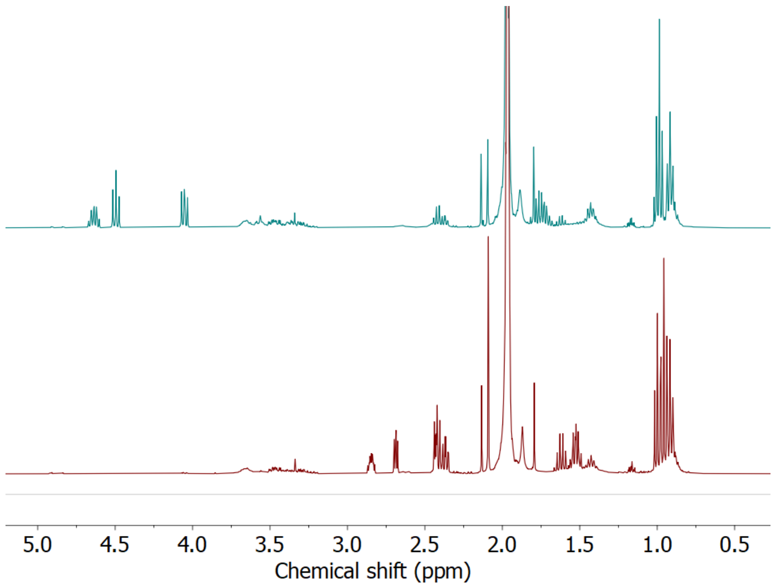

Figure S15: ^{1}H-NMR spectrum of supernatant of [EDH$^+$Br$^-$]-Ac-NH-Cr-BDC with EB in the absence of CO_2 for 26 h (bottom, red), reaction with EB in presence of 1 bar CO_2 for 26 h (top, teal).

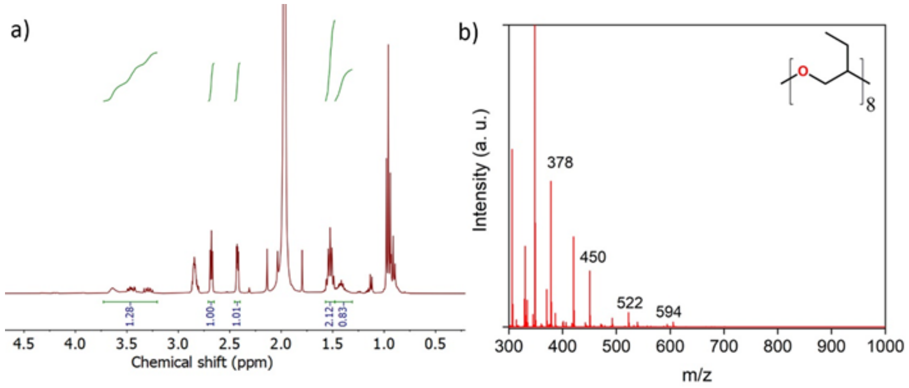

Figure S16: a) ^{1}H-NMR and b) MALDI-MS of supernatant of Cr-BDC MOF at the end of control reaction with EB in acetonitrile at 70 ºC for 26 h in the absence of CO_2.

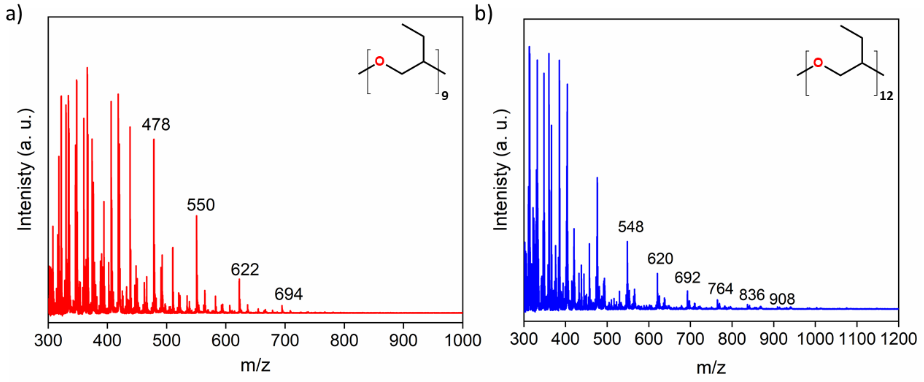

Figure S17: MALDI-MS of supernatant of a) NH_2-Cr-BDC and b) EDH^+Br^--Ac-NH-Cr-BDC at the end of control reaction with EB in the absence of CO_2

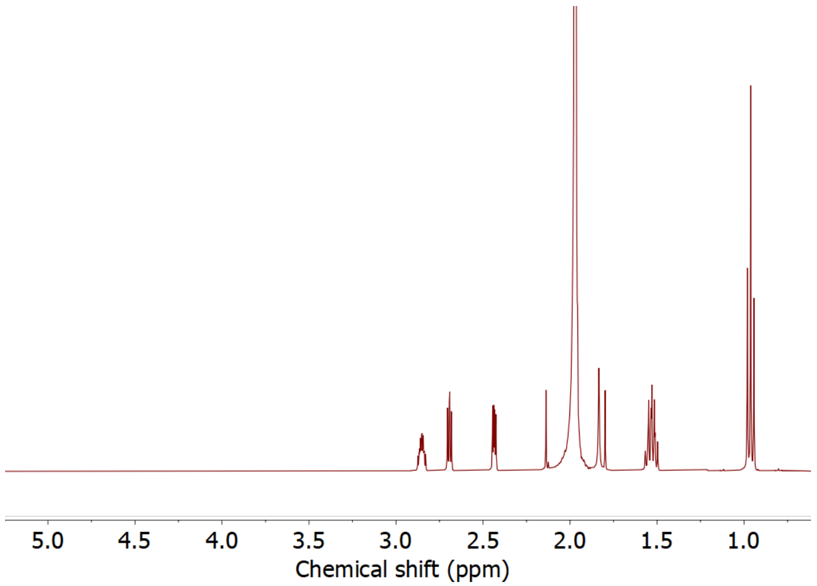

Figure S18: ^{1}H-NMR spectrum of EB in acetonitrile solvent in the absence of MOF and CO_2 for 26 h at 70 °C.

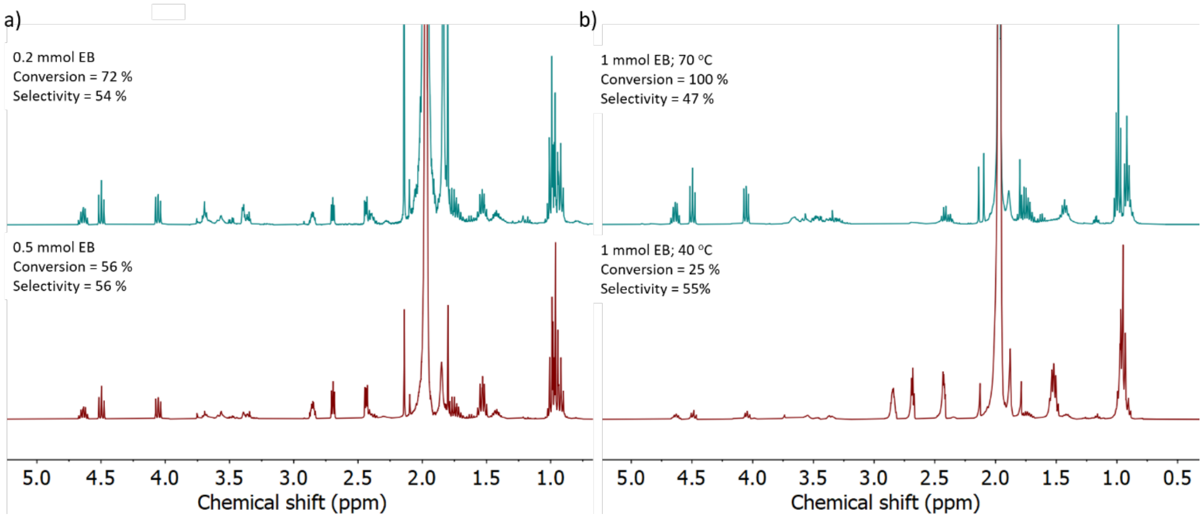

Figure S19: Control reactions on [EDH^+Br^-]-Ac-NH-Cr-BDC at 1 bar CO_2 and 70 °C for 26 h; a) reducing the concentration of EB to 0.5 mmol EB (maroon, bottom), 0.2 mmol EB (teal, top) and b) lowering the temperature from 70 °C (teal, top) to 40 °C (maroon, bottom).

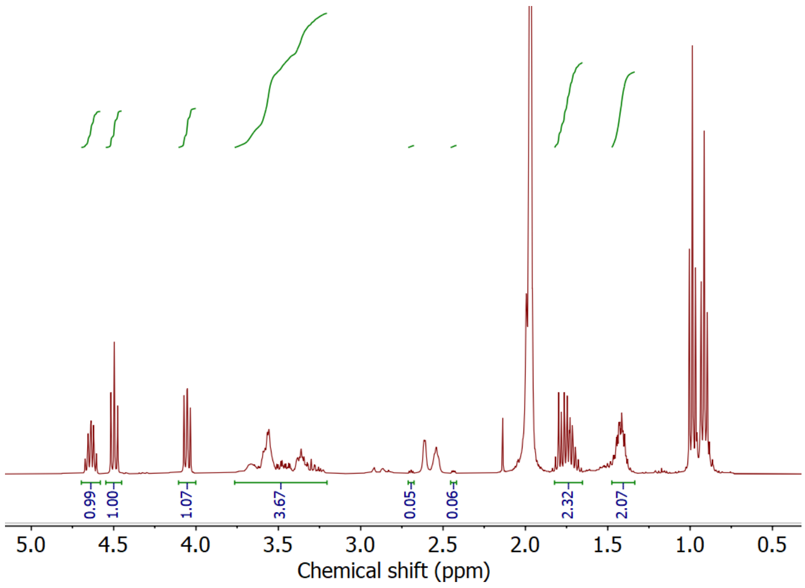

Figure S20: ^{1}H-NMR spectrum of supernatant of NH_2-Cr-BDC after catalysis in 5 bar CO_2 for 26 h.

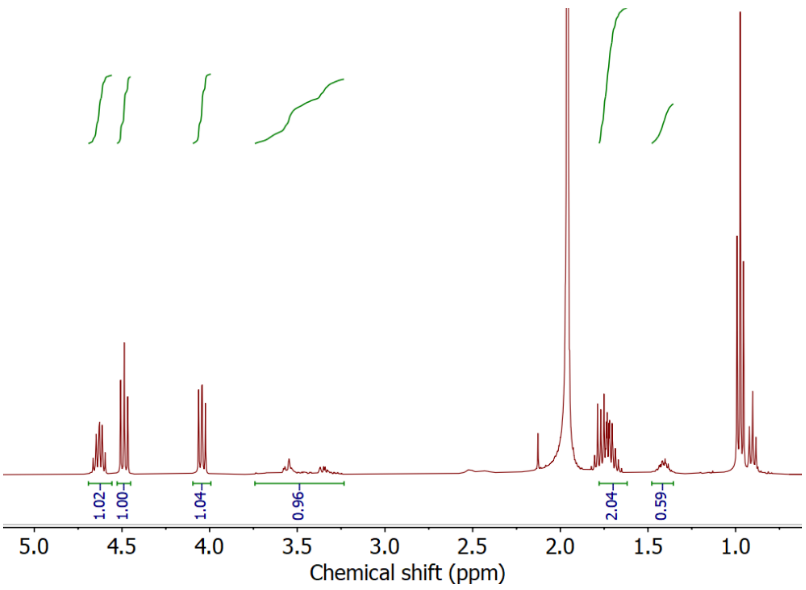

Figure S21: ^{1}H-NMR spectrum of supernatant of [EDH$^+$Br$^-$]-Ac-NH-Cr-BDC after catalysis in 5 bar CO_2 for 26 h.

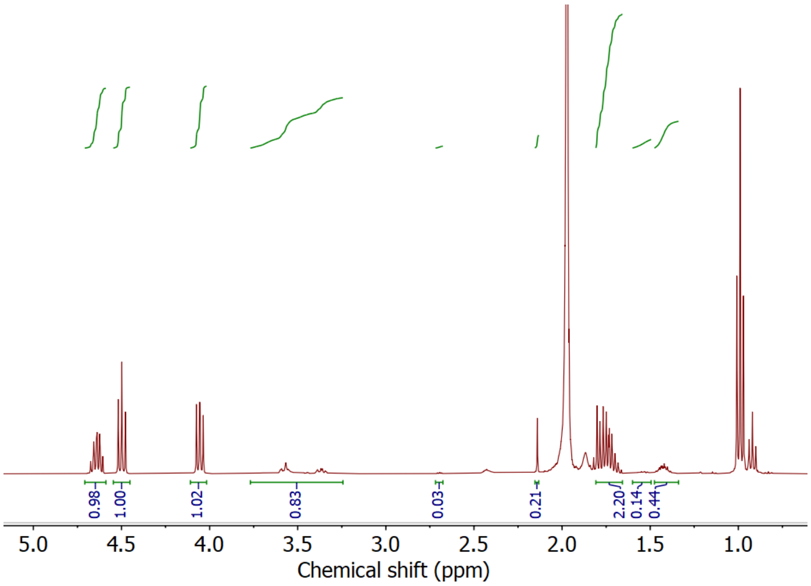

Figure S22: ^{1}H-NMR spectrum of supernatant of [DETAH$^+$Br$^-$]-Ac-NH-Cr-BDC after catalysis in 5 bar CO_2 for 26 h.

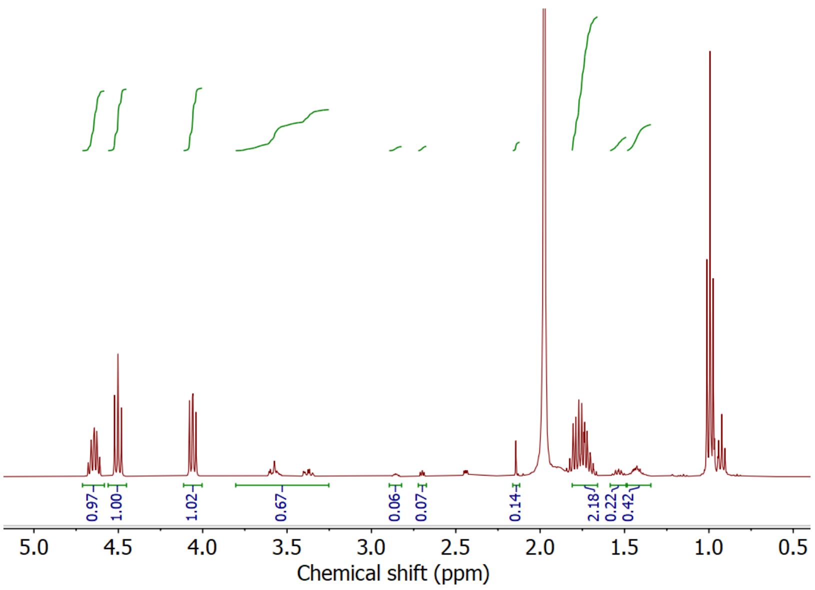

Figure S23: ^{1}H-NMR spectrum of supernatant of [TAEAH$^+$Br$^-$]-Ac-NH-Cr-BDC after catalysis in 5 bar CO_2 for 26 h.

Table S4: Catalytic performance of materials at 5 bar CO_2, 70 °C for 26 h.

Catalysts	Conversion (%)	Selectivity (%)
NH_2-Cr-BDC	99.2 (±1.38)	51.3 (±2.0)
[EDH$^+$Br$^-$]-Ac-NH-Cr-BDC	99.7 (±0.57)	77.2 (±2.02)
[DETAH$^+$Br$^-$]-Ac-NH-Cr-BDC	97.5 (±1.5)	82.7 (±0.68)
[TAEAH$^+$Br$^-$]-Ac-NH-Cr-BDC	93.8 (±0.45)	84.1 (±1.72)

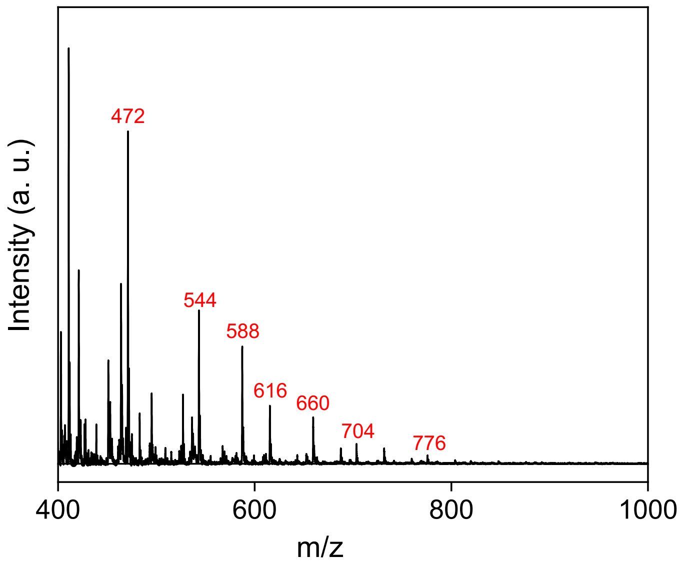

Figure S24: MALDI-MS of supernatant of NH_2-Cr-BDC after catalysis in 5 bar CO_2.

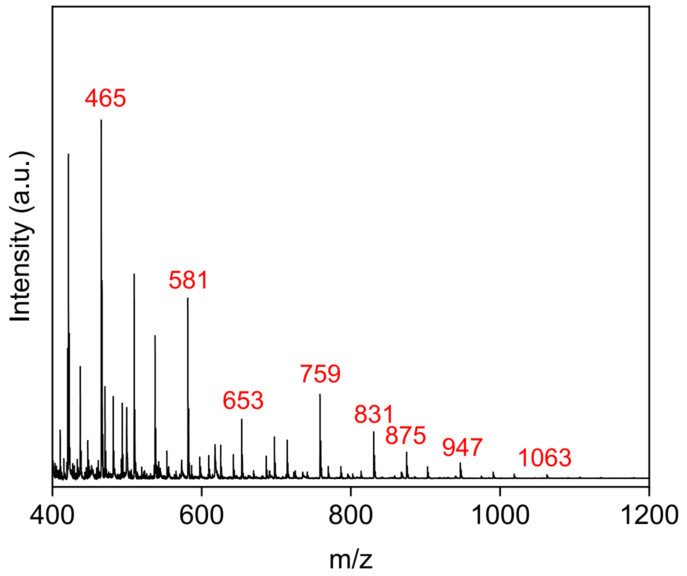

Figure S25: MALDI-MS of supernatant from [EDH$^+$Br$^-$]-Ac-NH-Cr-BDC after catalysis in 5 bar CO_2

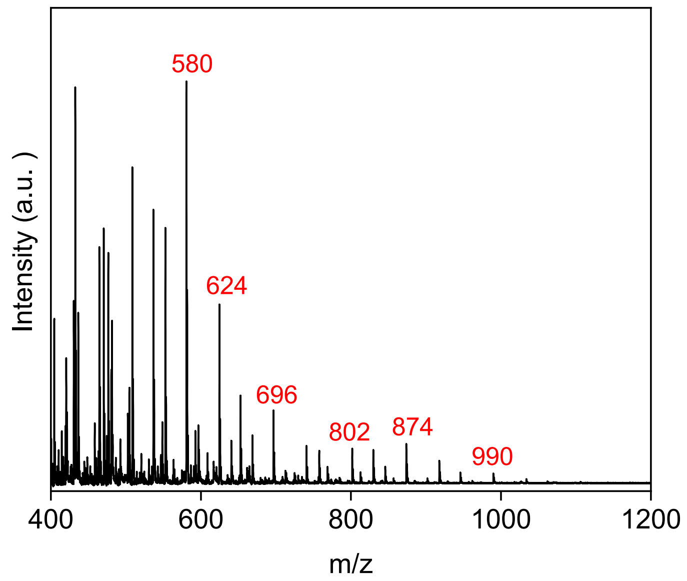

Figure S26: MALDI-MS of supernatant from [DETAH$^+$Br$^-$]-Ac-NH-Cr-BDC after catalysis in 5 bar CO_2

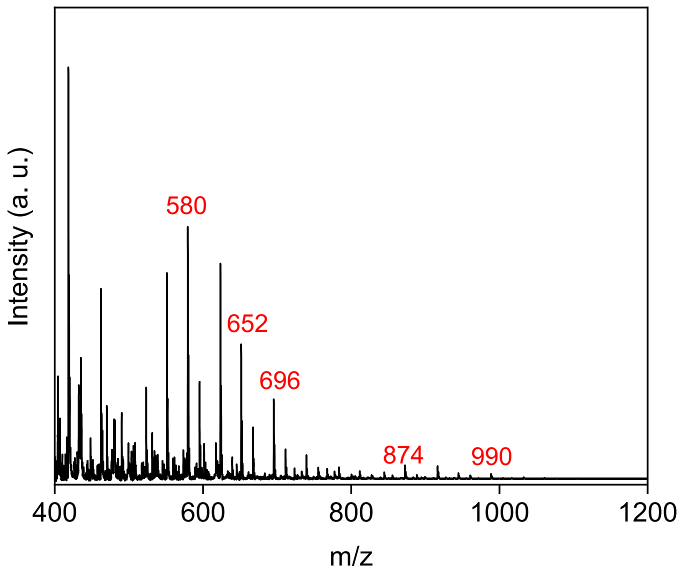

Figure S27: MALDI-MS of supernatant from [TAEAH$^+$Br$^-$]-Ac-NH-Cr-BDC after catalysis in 5 bar CO_2

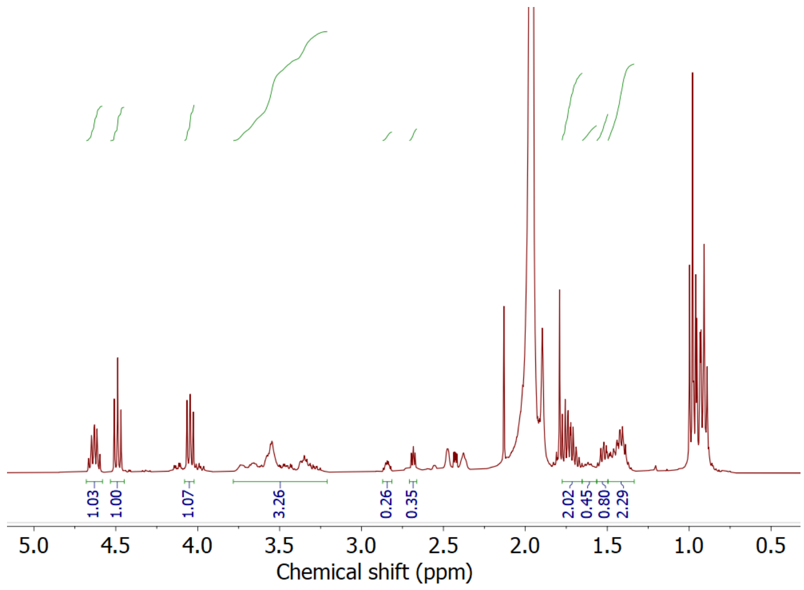

Figure S28: ^{1}H-NMR of cycle 1 of bare MOF, NH_2-Cr-BDC after catalysis in 5 bar CO_2 for 6 h.

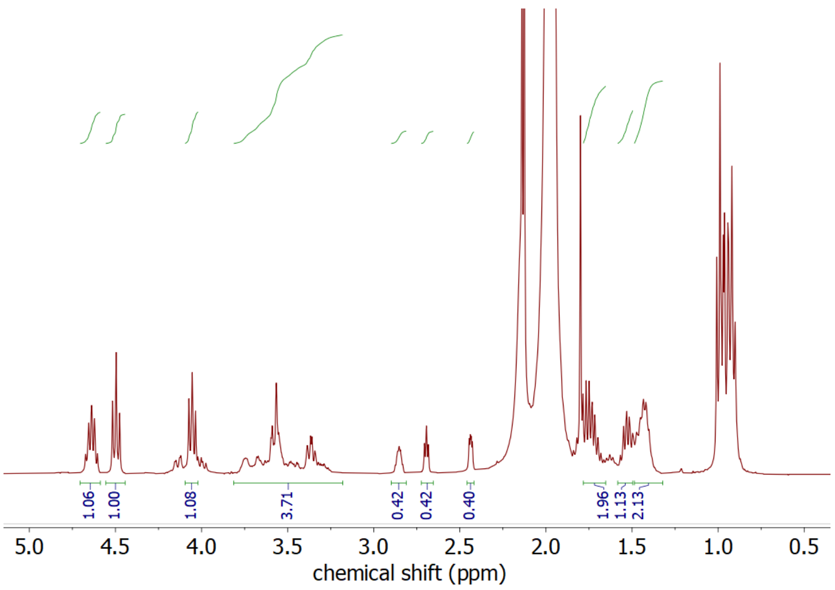

Figure S29: ^{1}H-NMR of cycle 2 of bare MOF, NH_2-Cr-BDC after catalysis in 5 bar CO_2 for 6 h.

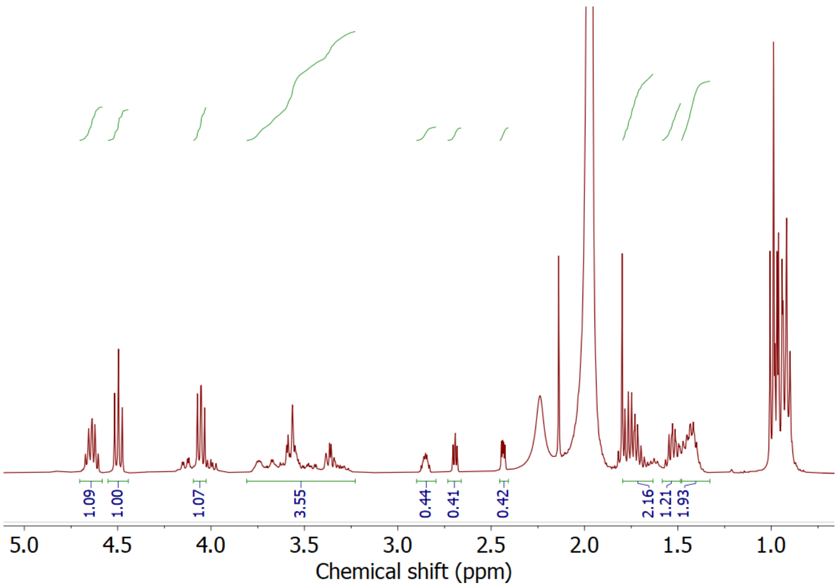

Figure S30: ^{1}H-NMR of cycle 3 of bare MOF, NH_2-Cr-BDC after catalysis in 5 bar CO_2 for 6 h.

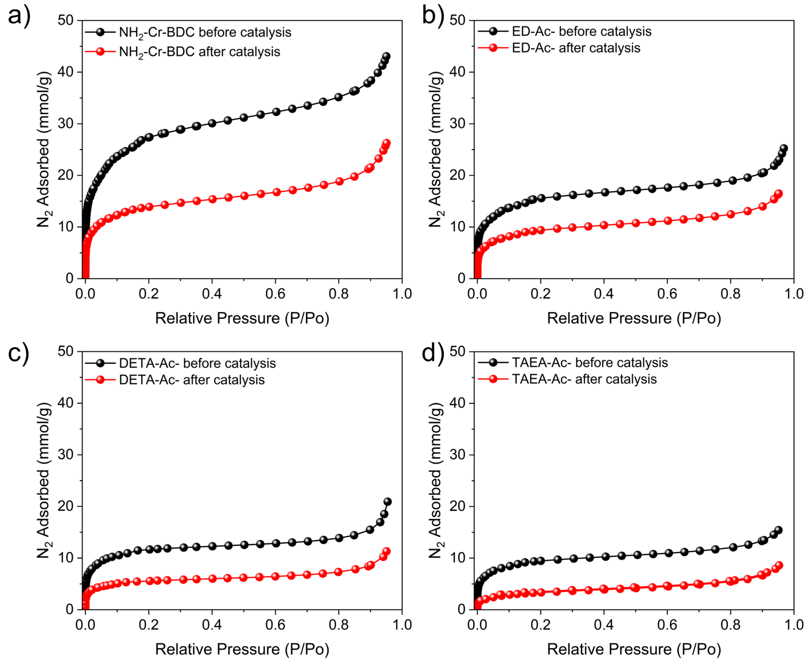

Figure S31: N_2 at 77 K of catalysts before and after catalysis in 5 bar CO_2, a) NH_2-Cr-BDC MOF, b) ED-Ac-NH-Cr-BDC, c) DETA-Ac-NH-Cr-BDC and d) TAEA-NH-Cr-BDC

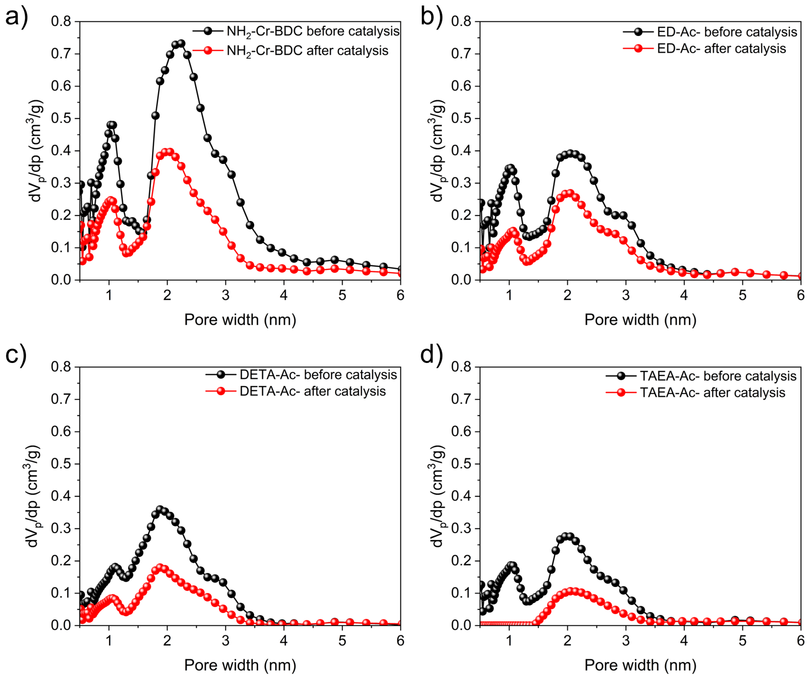

Figure S32: pore size distribution of catalysts before and after catalysis in 5 bar CO_2, a) NH_2-Cr-BDC MOF, b) ED-Ac-NH-Cr-BDC, c) DETA-Ac-NH-Cr-BDC and d) TAEA-NH-Cr-BDC. NLDFT methodology is used for the pore size distribution.

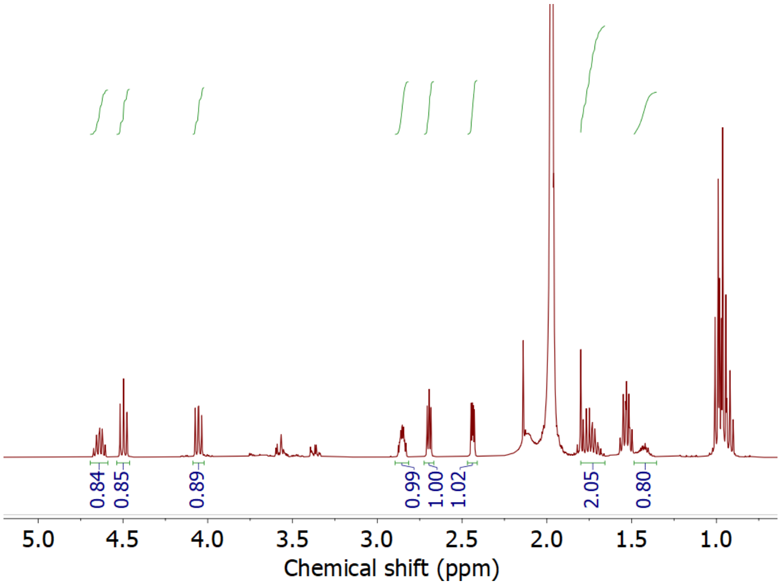

Figure S33: ^{1}H-NMR of cycle 1 of [EDH$^+$Br$^-$]-Ac-NH-Cr-BDC after catalysis in 5 bar CO_2 for 6 h.

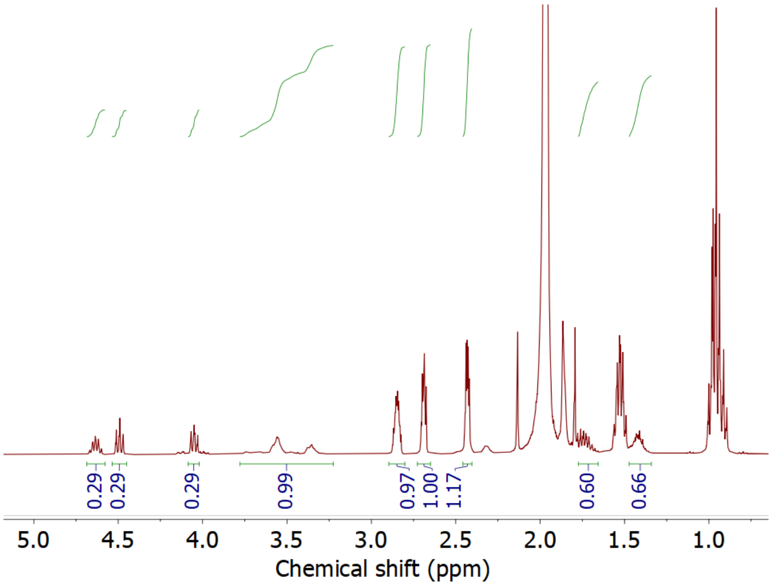

Figure S34: ^{1}H-NMR of cycle 2 of [EDH$^+$Br$^-$]-Ac-NH-Cr-BDC after catalysis in 5 bar CO_2 for 6 h.

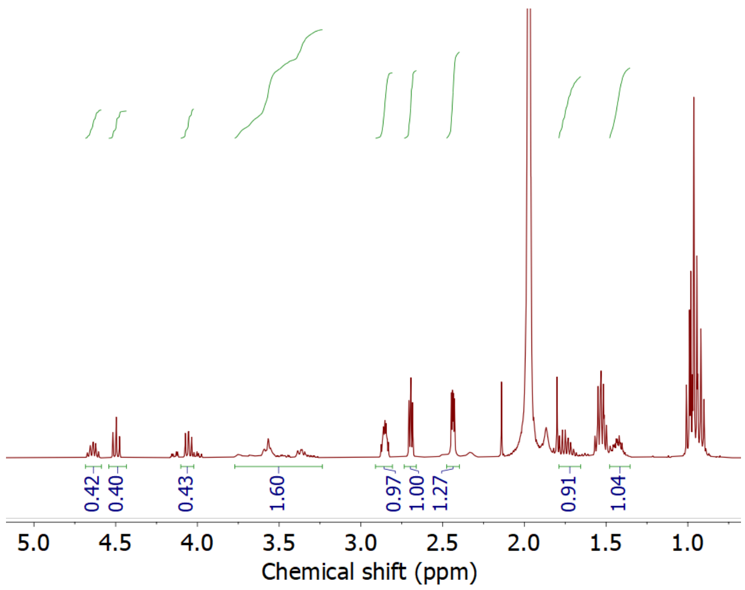

Figure S35: ^{1}H-NMR of cycle 3 of [EDH$^+$Br$^-$]-Ac-NH-Cr-BDC, after catalysis in 5 bar CO_2 for 6 h.

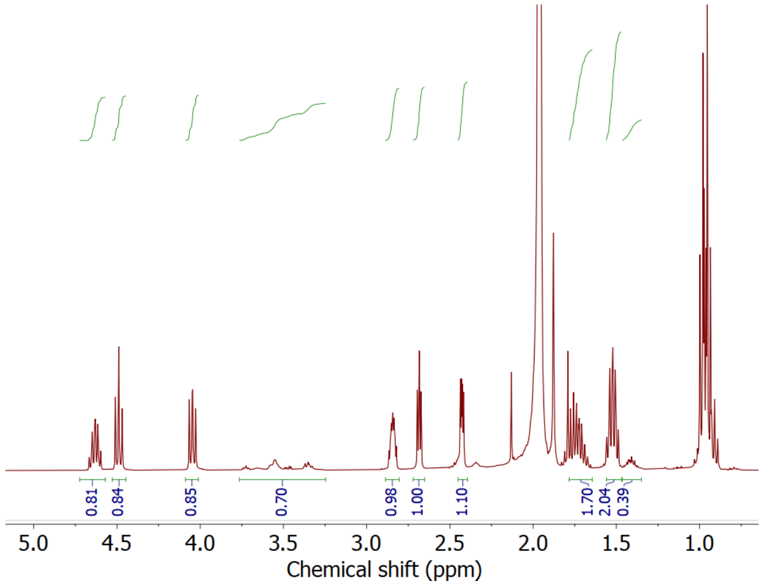

Figure S36: ^{1}H-NMR of cycle 1 of. [DETAH$^+$Br$^-$]-Ac-NH-Cr-BDC after catalysis in 5 bar CO_2 for 6 h.

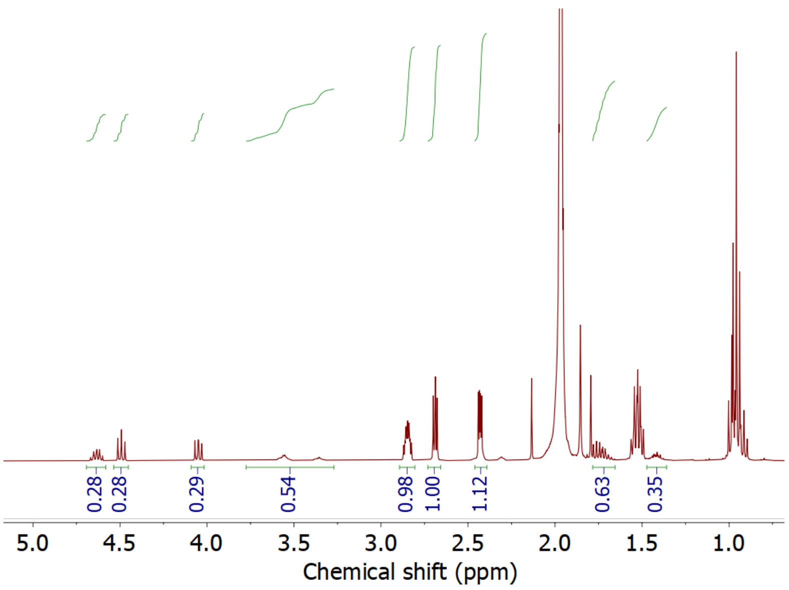

Figure S37: ^{1}H-NMR of cycle 2 of [DETAH$^+$Br$^-$]-Ac-NH-Cr-BDC after catalysis in 5 bar CO_2 for 6 h.

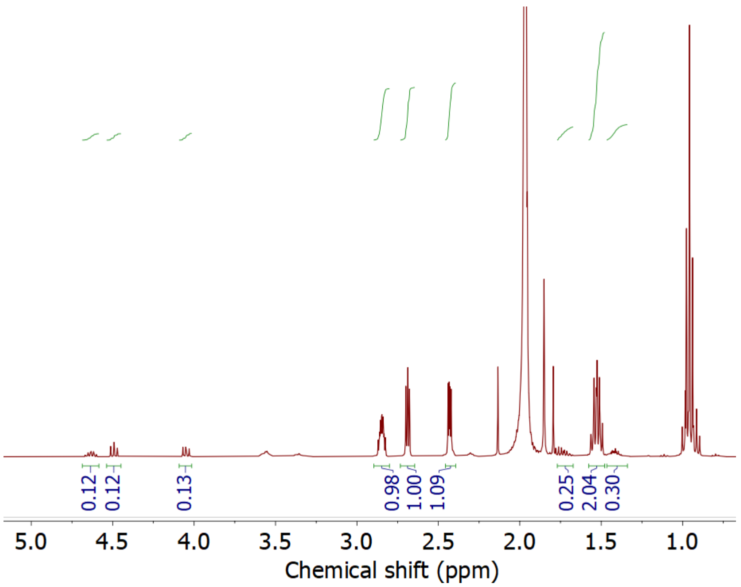

Figure S38: ^{1}H-NMR of cycle 3 of [DETAH$^+$Br$^-$]-Ac-NH-Cr-BDC after catalysis in 5 bar CO_2 for 6 h.

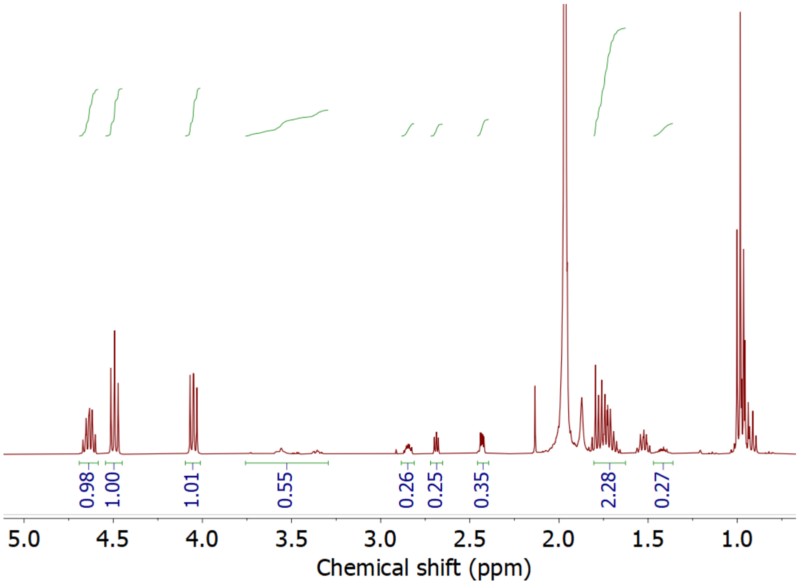

Figure S39: ^{1}H-NMR of cycle 1 of [TAEAH$^+$Br$^-$]-Ac-NH-Cr-BDC after catalysis in 5 bar CO_2 for 6 h.

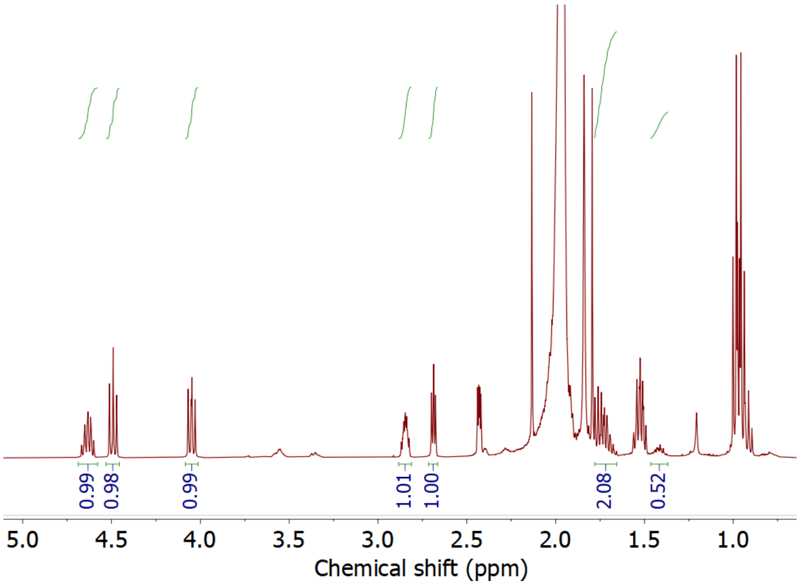

Figure S40: ^{1}H-NMR of cycle 2 of [TAEAH$^+$Br$^-$]-Ac-NH-Cr-BDC after catalysis in 5 bar CO_2 for 6 h.

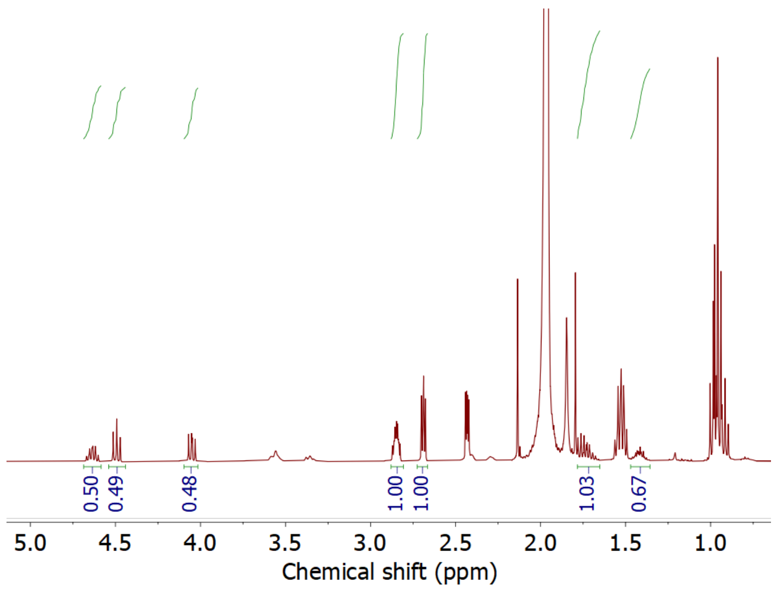

Figure S41: ^{1}H-NMR of cycle 3 of [TAEAH$^+$Br$^-$]-Ac-NH-Cr-BDC after catalysis in 5 bar CO_2 for 6 h.

Table S5: Reusability of the catalyst in 3 cycles in 5 bar CO_2, at 70 °C for 6 h.

NH_2-Cr-BDC	Conversion (%)	Selectivity (%)
cycle 1	89.63 (±3.56)	46.5 (±1.5)
cycle 2	83.33 (±0.57)	45.97 (±1.6)
cycle 3	83.5 (±3.27)	49.5 (±3.0)
[EDH$^+$Br$^-$]-Ac-NH-Cr-BDC		
cycle 1	44.47 (±5.8)	68.1 (±3.2)
cycle 2	40.4 (±2.4)	48.5 (±0.8)
cycle 3	45.3 (±2.46)	46.3 (±3.5)
[DETAH$^+$Br$^-$]-Ac-NH-Cr-BDC		
cycle-1	51.3 (±0.6)	80.77 (±0.68)
cycle-2	32.07 (±0.9)	62.9 (±1.3)
cycle-3	22.08 (±2.6)	45.7 (±0.57)
[TAEAH$^+$Br$^-$]-Ac-NH-Cr-BDC		
cycle 1	81.57 (±1.9)	88.5 (±0.78)
cycle 2	55.4 (±1.5)	78.77 (±1.57)
cycle 3	45.1 (±1.0)	59.27 (±1.25)

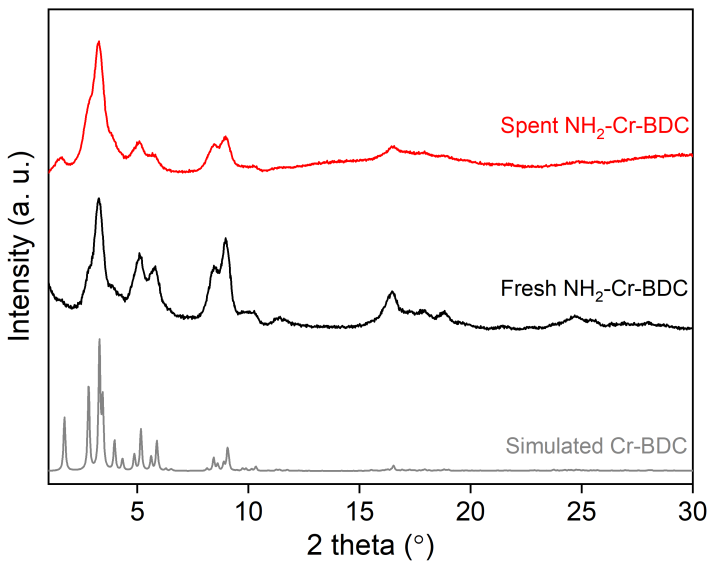

Figure S42: PXRD of simulated Cr-BDC MOF (grey), fresh NH_2-Cr-BDC (black) and spent catalyst after 3 cycles (red).

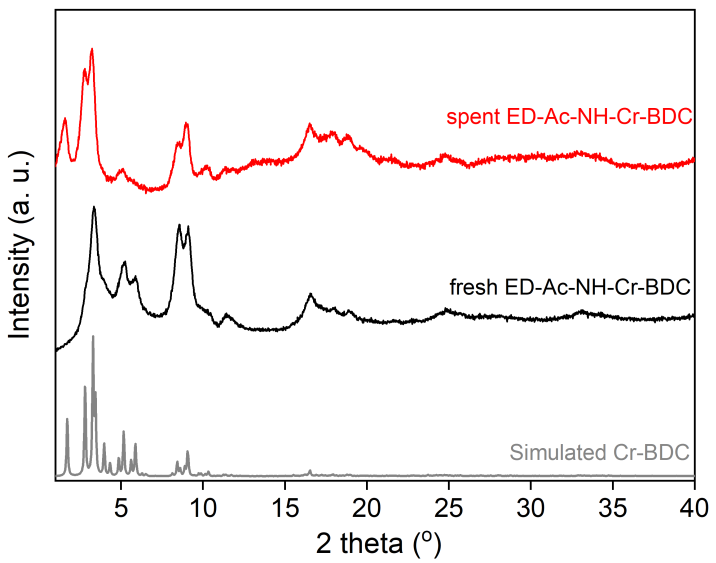

Figure S43: PXRD of simulated Cr-BDC MOF (grey), fresh spent [EDH^+Br^-]-Ac-NH-Cr-BDC (black) and spent catalyst (red).

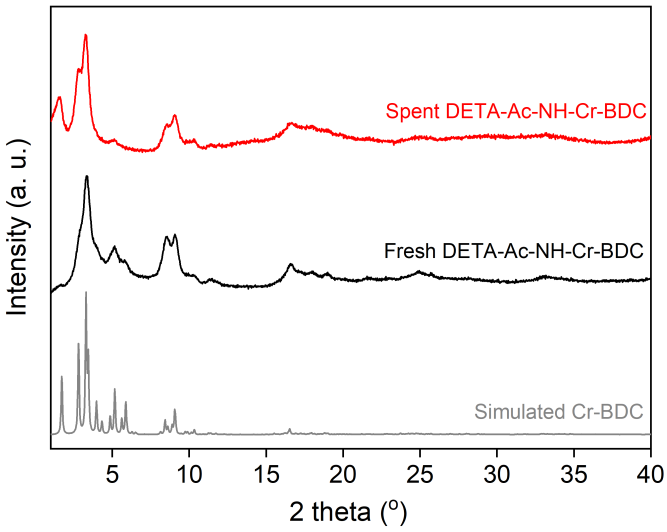

Figure S44: PXRD of simulated Cr-BDC MOF (grey), fresh [$DETAH^{+}Br^{-}$]-Ac-NH-Cr-BDC (black) and spent catalyst (red).

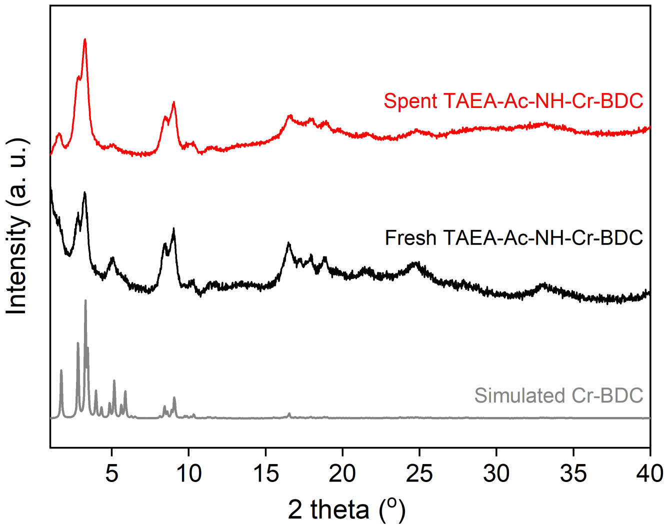

Figure S45: PXRD of simulated Cr-BDC MOF (grey), fresh [$TAEAH^{+}Br^{-}$]-Ac-NH-Cr-BDC (black) and spent catalyst (red).

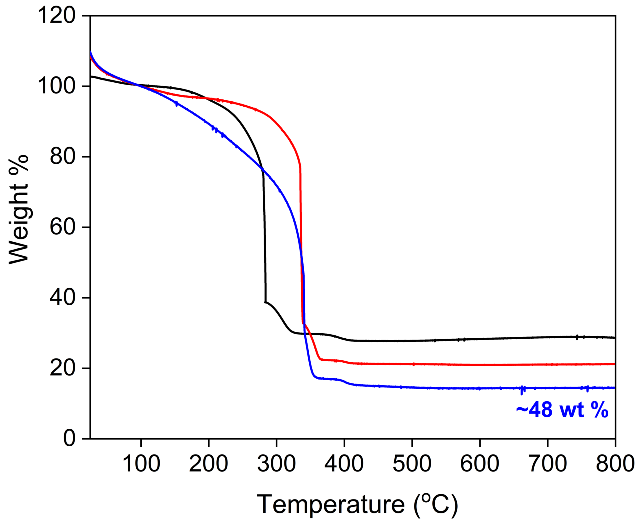

Figure S46: TGA of bare MOF (black), [EDH$^+$Br$^-$]-Ac-NH-Cr-BDC fresh catalyst (red) and spent catalyst (blue).

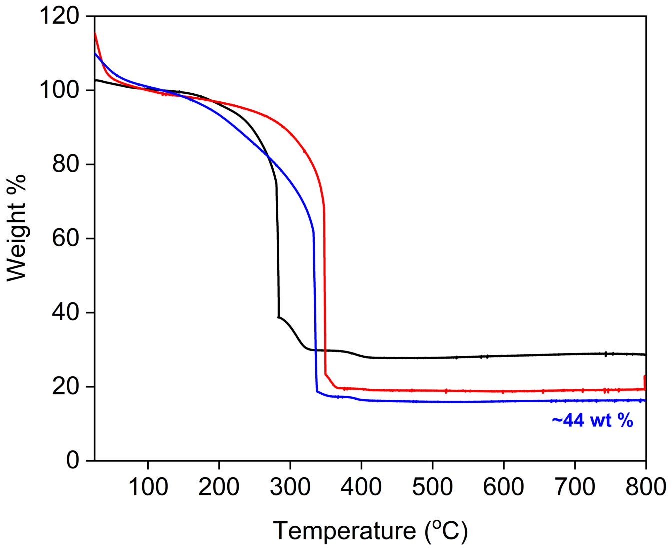

Figure S47: TGA of bare MOF (black), fresh [DETAH$^+$Br$^-$]-Ac-NH-Cr-BDC (red) and spent catalyst (blue).

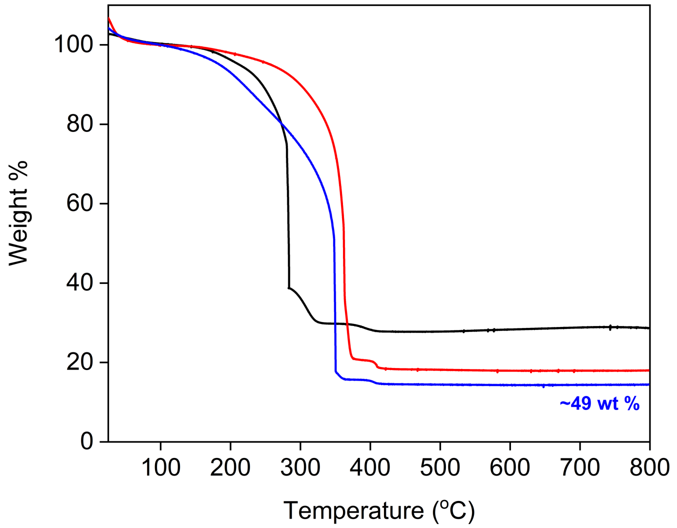

Figure S48: TGA of bare MOF (black), [TAEAH$^+$Br$^-$]-Ac-NH-Cr-BDC fresh catalyst (red) and spent catalyst (blue).

Table S6: Surface area, pore volume, Br- content and organic weight loading change in catalysts before and after catalysis in 5 bar CO_2.

Catalysts	SA before (m^2/g)	SA after (m^2/g)	% drop SA	V_p before (cm^3/g)	V_p after (cm^3/g)	% drop in V_p
NH_2-Cr-BDC	2200	1140	48.2	1.49	0.91	38.9
ED-Ac-	1260	765	39.3	0.87	0.57	34.4
DETA-Ac-	950	460	51.6	0.72	0.39	35.8
TAEA-Ac-	780	270	65.4	0.57	0.29	49.1
Catalysts	TGA before catalysis	TGA after 3 runs of catalysis	wt % increase in organics after catalysis	Br- before (wt %)	Br- after (wt %)	% drop in Br-
NH_2-Cr-BDC	0	22	22	-	-	
ED-Ac-	22	48	25	4.9	1.33	72.8
DETA-Ac-	32	44	15.8	5.2	1.9	63.4
TAEA-Ac-	35	49	17.7	5.1	1.44	71.7
